# Microbial polyphenol metabolism is part of the thawing permafrost carbon cycle

**DOI:** 10.1038/s41564-024-01691-0

**Published:** 2024-05-28

**Authors:** Bridget B. McGivern, Dylan R. Cronin, Jared B. Ellenbogen, Mikayla A. Borton, Eleanor L. Knutson, Viviana Freire-Zapata, John A. Bouranis, Lukas Bernhardt, Alma I. Hernandez, Rory M. Flynn, Reed Woyda, Alexandra B. Cory, Rachel M. Wilson, Jeffrey P. Chanton, Ben J. Woodcroft, Jessica G. Ernakovich, Malak M. Tfaily, Matthew B. Sullivan, Gene W. Tyson, Virginia I. Rich, Ann E. Hagerman, Kelly C. Wrighton

**Affiliations:** 1 Department of Soil and Crop Science, Colorado State University, Fort Collins, CO USA; 2 Department of Microbiology, The Ohio State University, Columbus, OH USA; 3 Center of Microbiome Science, The Ohio State University, Columbus, OH USA; 4 Department of Chemistry and Biochemistry, Miami University, Oxford, OH USA; 5 Department of Environmental Science; University of Arizona, Tucson, AZ USA; 6 Department of Natural Resources and the Environment, University of New Hampshire, Durham, NH USA; 7 Department of Environmental Sciences, Emory University, Atlanta, GA USA; 8 Department of Earth Ocean and Atmospheric Sciences, Florida State University, Tallahassee, FL USA; 9 Centre for Microbiome Research, School of Biomedical Sciences, Queensland University of Technology (QUT), Translational Research Institute, Woolloongabba, Queensland Australia

**Keywords:** Soil microbiology, Biogeochemistry

## Abstract

With rising global temperatures, permafrost carbon stores are vulnerable to microbial degradation. The enzyme latch theory states that polyphenols should accumulate in saturated peatlands due to diminished phenol oxidase activity, inhibiting resident microbes and promoting carbon stabilization. Pairing microbiome and geochemical measurements along a permafrost thaw-induced saturation gradient in Stordalen Mire, a model Arctic peatland, we confirmed a negative relationship between phenol oxidase expression and saturation but failed to support other trends predicted by the enzyme latch. To inventory alternative polyphenol removal strategies, we built CAMPER, a gene annotation tool leveraging polyphenol enzyme knowledge gleaned across microbial ecosystems. Applying CAMPER to genome-resolved metatranscriptomes, we identified genes for diverse polyphenol-active enzymes expressed by various microbial lineages under a range of redox conditions. This shifts the paradigm that polyphenols stabilize carbon in saturated soils and highlights the need to consider both oxic and anoxic polyphenol metabolisms to understand carbon cycling in changing ecosystems.

## Abstract (web summary)

Diverse microbial polyphenol transformations in thawing permafrost refute the theory that these compounds stabilize soil carbon across Arctic landscapes.

## Main

Permafrost stores an estimated 50% of global soil carbon^1,2^, nearly twice the amount of carbon in the atmosphere^3^. Increasing global temperatures are thawing permafrost and risk destabilizing this carbon^4^. Newly thawed carbon may be decomposed by resident microbial communities, yielding carbon dioxide (CO_2_) or methane (CH_4_), greenhouse gases that can accelerate climate warming^5,6^. Therefore, understanding microbial carbon processing in these climatically vulnerable habitats is critical to accurately project global warming feedbacks.

For decades, soil carbon stability in these systems was thought to be governed by the enzyme latch theories^7–9^ which propose that polyphenols inhibit microbial decomposition under anoxic conditions to ‘dead end’ soil carbon cycling. Polyphenols are a chemically diverse and abundant^10^ group of plant-derived compounds, spanning more than 10,000 chemical formulae and several structural families (Supplementary Fig. 1), including polymers (for example, tannins), monomers (for example, flavonoids) and simple phenols (for example, phenolic acids)^11^. Despite this vast chemical diversity, the enzyme latch theory recognizes oxygen-requiring phenol oxidases (POs) as being solely responsible for eliminating polyphenolic and phenolic compounds^7,9^ (Supplementary Note 1). Under this theory, saturated soil conditions (such as those post permafrost thaw) would render POs inactive, resulting in accumulated polyphenols that restrict microbial carbon decomposition, ultimately halting microbial soil carbon decomposition to stabilize soil carbon. Several studies have found conflicting support for parts of the enzyme latch theory^12–15^ or provided alternative biogeochemical explanations for results in the cascade^16^. However, even in these refuting studies, the primacy of POs in polyphenol degradation was taken for granted, leaving the possibility of alternative microbial metabolic strategies unexplored.

The microbial assumptions underlying the enzyme latch theory are not aligned with concepts from modern microbiome science. First, plants have been producing polyphenols for hundreds of millions of years^17,18^. Therefore, it is likely that diverse mechanisms for utilization, resistance and tolerance have evolved in microbial communities often exposed to polyphenols^19^. Second, the assumption that a single enzyme type transforms a broad class of compounds, and under strict environmental conditions, contrasts examples of enzyme specificity at environmental^20^, compound^21^ and even stereochemical levels^22^. Indeed, biochemical investigations across ecosystems such as the human gut have uncovered multiple non-PO microbial enzymes that degrade diverse polyphenols, including examples under anoxia^23^. Critically, the use of these enzymes by peatland and even terrestrial microbiomes has yet to be demonstrated in situ.

Here we sought to revisit the fundamental assumptions of the enzyme latch theory and to more broadly describe microbial polyphenol transformations expressed in peatland soils. We selected a model Arctic permafrost peatland, Stordalen Mire^5,24^, where the enzyme latch theory has been suggested to mediate carbon storage^25–27^. At this site, natural thaw created three distinct habitats from dry, intact permafrost palsa through a partially thawed bog with a fluctuating water table, to a fully thawed and saturated fen (Supplementary Fig. 2). To track microbial polyphenol metabolism across this thaw gradient, we obtained paired genome-resolved metatranscriptome, metabolite and geochemical data from peat cores taken from the palsa, bog and fen in July 2016. Leveraging this dataset, we provide a revised view of polyphenols in microbially catalysed carbon cycling in these climate-threatened habitats.

## Results

### Challenges to the enzyme latch in Stordalen Mire

**Fig. 1:**
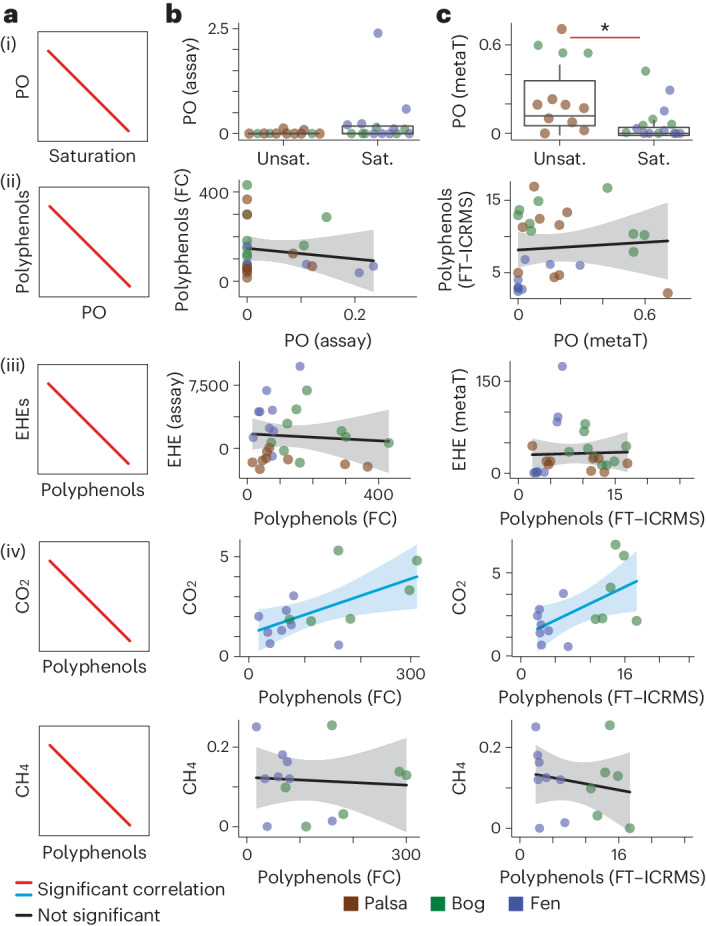
Multimethod investigation of the enzyme latch theory. **a**, The expected relationships proposed by the enzyme latch theory, with relationships labelled (**i**)–(**iv**) as in the text. **b**,**c**, The matched observed relationships using ‘Traditional’ assay methods (**b**) versus ‘omics and high-resolution methods (**c**). In **b**(**i**) and **c**(**i**), the lower and upper boxplot edges represent the 25th and 75th percentiles, respectively, and the middle line is the median. The whiskers extend from the median to 1.5× the interquartile range. *P = 0.005848, Wilcoxon rank-sum test. In **b**(**ii**)–(**iv**) and **c**(**ii**)–(**iv**), the linear trendlines for significant Pearson correlations (Benjamini–Hochberg *P*_adj_ < 0.05) are coloured blue (positive correlation) and red (negative correlation). The shaded regions correspond to the 95% confidence intervals. Water saturation is used as a proxy for oxygen availability, with unsaturated (Unsat.) corresponding to high oxygen, and saturation (Sat.) corresponding to low/no oxygen. Phenol oxidase activity is given by phenol oxidase assay (PO assay, nmol activity g^−1^ h^−1^) and the summed metatranscriptome expression of phenol oxidase genes from MAGs in the metatranscriptome (see Methods, PO metaT, geTMM). Polyphenol content is given by Folin–Ciocalteu phenolics (FC polyphenols, mg methyl-gallate equivalents per dry g soil) and polyphenol-like compounds identified in FT-ICRMS (% polyphenols; Supplementary Fig. 11). Extracellular hydrolase enzymes (EHE) are given by beta-glucosidase activity (EHE assay, nmol activity g^−1^ h^−1^) and the summed expression of genes encoding glycoside hydrolases from MAGs in the metatranscriptomes (EHE metaT, geTMM). This is representative of relationships observed for other enzyme types (see Supplementary Fig. 3). Porewater CO_2_ and CH_4_ are given in concentrations (mM). Individual correlation coefficients, *P* values and sample sizes are given in Supplementary Data 1.

First, we wanted to establish whether relationships put forth in the enzyme latch theories held true in Stordalen Mire. If an enzyme latch was controlling microbial carbon cycling, we expected to observe: (1) a negative relationship between PO expression and water saturation, (2) a negative relationship between PO expression and polyphenol concentrations, (3) a negative relationship between polyphenols and extracellular hydrolase enzymes and (4) a negative relationship between polyphenols and porewater CO_2_ and CH_4_ (Fig. 1a). We assessed these variables with the traditional methods used to support the enzyme latch theory^7,12^. To ensure that interpretation was not hampered by methodological constraints, we paired the traditional methods with high-resolution, multi-omic microbiome methods.

To test the first prediction, we analysed differences in assayed PO activity and metatranscriptome expression of PO genes between unsaturated and saturated peat samples. Using a traditional phenol oxidase assay, we saw no significant difference in PO activity between unsaturated and saturated samples (Fig. 1b(i) and Supplementary Data 1). However, from the metatranscriptome data, we detected significantly less PO gene expression in saturated samples than in unsaturated samples, mirroring the expected relationship of these oxygen-requiring genes (Fig. 1c(i) and Supplementary Data 1). These conflicting results between traditional enzyme assay and our field gene expression data could be due to the convention of conducting the PO assay under oxic conditions in the laboratory instead of maintaining the field relevant redox conditions^12^, suggesting that metatranscriptome expression is a more sensitive measure of PO expression under field conditions.

For the second prediction, in contrast to theoretical expectations, we did not observe a significant negative relationship between POs and polyphenol concentrations using either traditional or multi-omic methods (Fig. 1(bii),c(ii) and Supplementary Data 1). Similarly, there were no correlations between polyphenols and assayed enzyme activity or glycoside hydrolase metatranscriptome gene expression (Fig. 1b(iii),c(iii), and Supplementary Fig. 3 and Data 1). Lastly and notably, in contrast to the primary tenet of the enzyme latch theory, we found that polyphenol abundance was positively correlated with porewater CO_2_ (although not CH_4_) concentrations, indicating that polyphenol metabolism could contribute to peatland soil respiration (Fig. 1b(iv),c(iv) and Supplementary Data 1).

Collectively, traditional and multi-omics methods failed to support relationships expected by the enzyme latch theory at key steps in the biogeochemical cascade. This was consistent whether Stordalen Mire’s peatlands were assessed collectively (Fig. 1) or by habitat (that is, palsa, bog and fen; Supplementary Fig. 4 and Data 1), suggesting that the enzyme latch theory is not a suitable descriptor of this peatland’s carbon processing dynamics at any scale. Further, the lack of relationship between polyphenol oxidases and polyphenol content suggests that POs alone do not control the fate of these compounds along this thaw gradient. Given this and the positive relationship observed between polyphenol abundance and porewater CO_2_ concentrations, our data indicate that polyphenols do not inhibit microbial carbon metabolism and may even contribute to respiration in situ.

### Polyphenol transformations expressed across Stordalen Mire

We next sought to investigate the avenues of microbial polyphenol metabolism active in Stordalen Mire. Although the enzyme latch theory has long suggested that POs were the sole microbial enzymes controlling polyphenol degradation in soil systems^7^, biochemical investigations across microbiomes from diverse ecosystems have biochemically characterized microbial enzymes that act upon distinct polyphenol types, under a range of redox conditions and for diverse metabolic ends^23,28^. Unfortunately, these annotations were not translated into annotation frameworks, thus impeding their broader extension to other microbiomes.

Leveraging these studies, we developed an open-source annotation tool called CAMPER (Curated Annotations for Microbial Polyphenol Enzymes and Reactions) that annotates and summarizes polyphenol transformation potential from assembled gene data^29^. This tool searches 41 custom (Supplementary Table 1) and 234 database-derived homology-based gene family annotations against user input sequence files. CAMPER assigns genes to a substrate ontology on the basis of established polyphenol chemical classes^30^ as well as aggregates this content into 100 transformation pathways (Supplementary Data 2). In addition, CAMPER pathways were coded for their oxygen dependence on the basis of previous biochemical knowledge. CAMPER pathways were also placed into a trophic degradation hierarchy on the basis of their transformation of polymeric polyphenols, monomeric polyphenols, or simple phenols. Finally, any additional information needed to manually curate the CAMPER annotations is provided, including gene cluster organization and expected secretion status. CAMPER compiles existing biochemical knowledge into a chemical framework to make polyphenol metabolisms more accessible to microbiome scientists.

**Fig. 2:**
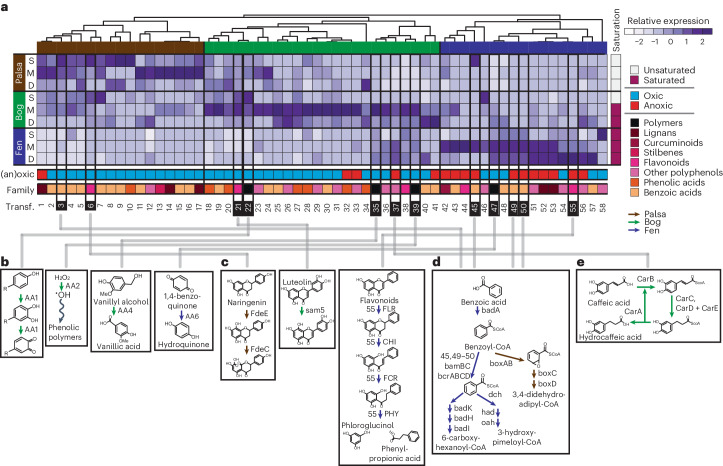
Polyphenol transformations expressed in Stordalen Mire. **a**, Hierarchical clustering of the relative metatranscriptome expression of polyphenol transformations across habitats and depths (rows: S, surface; M, middle; D, deep depths). Here, relative metatranscriptome expression is displayed as the *z*-score of the average of the summed expression (*n* = 3, geTMM) of genes in each pathway (columns). The saturation column to the right of the heat map denotes samples as saturated (red) if they were at or below the water table depth, or unsaturated (white) if they were above the water table (Supplementary Data 1 and Fig. 2). Hierarchical clustering of pathways revealed palsa (brown), bog (green) and fen (blue) specific transformations, indicated by top-row colour. The oxygen requirements for transformations are shown in the second bottom-most row for oxic (blue) and anoxic (red) transformations. The family of polyphenols that each pathway acts upon is coloured at the bottom-most row: black (polymers), pink shades (monomers) and orange shades (phenolic/benzoic acids). Column numbers correspond to transformations; see Supplementary Data 2 for more detail on each transformation. Transformations mentioned in the text are enclosed in a black box, and the numbers are highlighted black in the heat map to match the text reference, and the reactions are shown in **b**–**e**. Arrow colours correspond to the habitat cluster for the transformation: palsa (brown), bog (green) and fen (blue). In no. 39, the wavy arrow indicates that the hydroxyl radical diffuses away to act on phenolic polymers. All pathways are shown in Supplementary Fig. 6.

To identify potential polyphenol transformation strategies used by the Stordalen Mire microbiome, we used CAMPER to annotate a database of 1,864 metagenome-assembled genomes (MAGs) recovered from field-derived metagenomes taken over 8 years across the site (Supplementary Data 2). We used this genomic framework to contextualize a metatranscriptome and metabolome dataset derived from triplicate palsa, bog and fen cores taken from July 2016, with cores divided at three depths (Supplementary Data 3 and 4). As preliminary evidence for polyphenol transformations in these soils, metabolomics (liquid chromatography with tandem mass spectrometry (LC–MS/MS)) identified 35 polyphenols across six families. In further support of active polyphenol metabolisms in situ, the metatranscriptome data uncovered 58 expressed polyphenol transformation pathways from across seven polyphenol families (Fig. 2, and Supplementary Figs. 5 and 6). Four of the detected polyphenol metabolites were substrates or products in expressed CAMPER pathways (Supplementary Fig. 5). We observed habitat- and depth-specific metabolite and gene expression patterns, probably due to differences in plant and microbial community species between habitats^5,31^. Importantly, unlike PO alone (Fig. 1b(ii),c(ii)), the collective expression of these 58 metatranscriptome pathways was significantly and positively related to polyphenol content (Supplementary Fig. 7). These findings illustrate the microbial metabolic plasticity overlooked by the enzyme latch theory which focuses on a single enzymatic type.

Due to varying water saturation conditions at the time of sampling (with all palsa depths and surface bog being dry, and deeper bog and fen being water saturated), we expected to observe an associated variation in oxic and anoxic polyphenol-transformation gene expression (Fig. 2a and Supplementary Fig. 2). In accordance with water table depth across these sites, the relative expression of genes encoding enzymes for anoxic polyphenol transformations was significantly higher in the saturated samples (Supplementary Fig. 7). This contrasts with the enzyme latch theory, as waterlogging does not appear to turn off microbial polyphenol metabolism.

Next, we inventoried polyphenol metabolism across the degradation hierarchy (polymeric polyphenol, monomeric polyphenol and phenolic/benzoic transformations). For polymers, genes encoding PO (AA1) were most enriched in bog surface-layer metatranscriptomes, consistent with the enzyme’s oxygen dependence (Fig. 2a no. 22 and Fig. 2b). In addition, we detected expression of genes encoding three non-PO enzymes reported to depolymerize polymeric polyphenols: peroxidase (AA2, Fig. 2a no. 39 and Fig. 2b), vanillyl-alcohol oxidase (AA4, Fig. 2a no. 35 and Fig. 2b) and benzoquinone reductase (AA6, Fig. 2a no. 47 and Fig. 2b)^32–34^. While AA2 and AA4 expression was enriched in the unsaturated bog samples, AA6 expression was enriched in the saturated fen samples and increased with depth (Fig. 2a). As bacterial AA6 enzymes have been suggested to function in anoxic polyphenol depolymerization^34^, their expression in the fen further refutes the enzyme latch assumption that polyphenol degradation is halted under water-saturated conditions. Rather, our data illustrate that peatland microbiomes express genes for multiple enzymes beyond phenol oxidases that operate across a range of redox conditions.

For monomeric polyphenols and phenolic/benzoic acids, we observed a trend similar to that of polymers where at a polyphenol family level, distinct transformation pathways were expressed depending on the redox environments. For example, the oxic pathways for flavonoids were enriched in the palsa and bog (Fig. 2a nos. 6 and 21 and Fig. 2c), and the anoxic flavonoid pathway was enriched in the water-saturated fen (Fig. 2a no. 55 and Fig. 2c). Furthermore, the oxic degradation pathway for benzoic acid was enriched in the palsa (Fig. 2a no. 3 and Fig. 2d), while three anoxic degradation pathways were enriched in the fen (Fig. 2a nos. 45, 49 and 50 and Fig. 2d). In all, this is important because these distinct transformation pathways associated with varying water saturation across the site lead to different metabolic outcomes. Taken together, we put forward the idea that redox state is an important environmental filter, not an ‘on/off’ switch, that alters the fate and metabolic consequences of polyphenols.

Our data further illuminated the myriad ways that polyphenols can contribute to microbial metabolism. Beyond supporting microbial metabolism as an electron donor or carbon source, the bog-enriched caffeic acid reductase system instead uses the polyphenol caffeic acid to accept electrons (Fig. 2a no. 37 and Fig. 2e)^35^. In this pathway, caffeic acid is activated to caffeyl-CoA and subsequently reduced to hydrocaffeyl-CoA, which transfers the CoA to another caffeic acid in an energy-saving loop. The reduction step generates NAD^+^ and reduces ferredoxin, which fuel the Rnf complex and a hydrogenase^28^. Interestingly, caffeic acid metabolite abundance is positively related to caffeic acid reductase gene expression in the fen (Supplementary Fig. 5), providing additional evidence that this metabolism may be occurring. This metabolism expands the role of polyphenols in soil microbiomes to alternative energy-generating mechanisms.

Collectively, our metatranscriptome data paint a complex view of microbiome–polyphenol interactions in peatlands, supporting the importance of polyphenols to peatland carbon cycling despite being historically overlooked as substrates. While the enzyme latch theory posited that strict redox conditions limit polyphenol transformations in peat, we revealed the potential for redox-tunable transformation strategies that enable microorganisms to use polyphenols for diverse metabolic ends.

### Diverse and talented polyphenol-active lineages

**Fig. 3:**
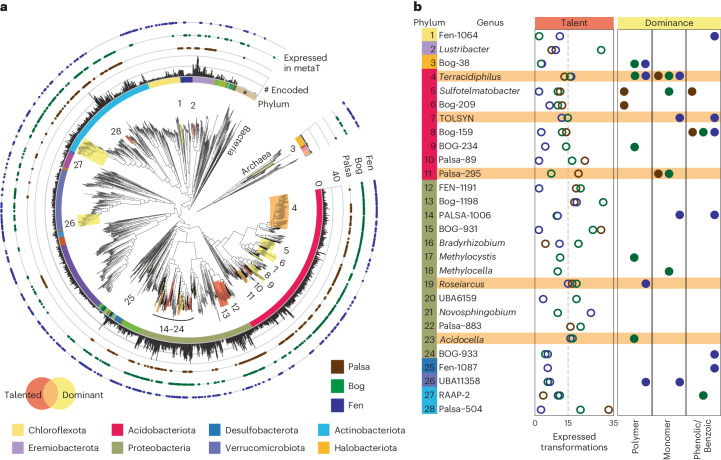
Polyphenol transformation potential encoded and expressed across Stordalen Mire MAGs. **a**, Phylogenetic trees of the 1,864 Stordalen Mire MAGs constructed using the GTDB 120 bacterial and 53 archaeal gene sets^53^, with the Patescibacteria and Micrarchaeota as outgroups, respectively. The inner multicoloured ring corresponds to phylum, with the Patescibacteria (in beige) indicated by an asterisk (*). The middle ring displays a bar chart of the number of polyphenol transformations encoded by each MAG (range 0–40). The outer rings indicate whether a given MAG expressed at least one polyphenol transformation in the palsa, bog or fen metatranscriptomes by the presence of a dot. Clades of ‘polyphenol talented’ genera are highlighted in red, polyphenol dominant genera are highlighted in yellow and genera that meet both definitions are highlighted in orange (bottom left legend). Numbering at clade tips corresponds to genus names in **b**. **b**, Plots showing the number of unique polyphenol transformation pathways expressed per genus in each habitat (left), and whether that genus contributed at least 10% of polymer, monomer or phenolic/benzoic acid transformations in any habitat (right). A dashed line at 15 expressed transformations delineates talented genera (Supplementary Fig. 10). Genera that are both talented and dominant are highlighted in orange.

Next, we wanted to catalogue the microorganisms encoding and expressing these polyphenol transformations. Illustrating the prevalence of these transformations in the thawed permafrost microbiome, only one of the 43 phyla captured by the 1,864 MAGs lacked appreciable polyphenol or subsequent phenolic transformations (the 58 Patescibacteria MAGs, Fig. 3a). MAGs from the Acidobacteriota, Proteobacteria, Actinobacteriota and Eremiobacteriota were particularly enriched in polyphenol metabolism, encoding on average 10 transformations per genome (Fig. 3a and Supplementary Fig. 8). Further, expression of these transformations spanned MAGs across the phylogenetic tree, in all three habitats (Fig. 3a and Supplementary Fig. 9).

Considering the broad phylogenetic range of the MAGs encoding and expressing these transformations, we next identified the important polyphenol-active lineages in Stordalen Mire. To do this, we defined two metrics of importance derived from the metatranscriptome data: (1) polyphenol talent, the number of different pathways expressed and (2) polyphenol dominance, the relative contribution to active polyphenol metabolism in Stordalen Mire (see Methods and Supplementary Fig. 10). We assessed these metrics for MAGs aggregated at the genus level (*n* = 302 genera) and identified 50 microbial genera spanning 11 phyla that exhibited polyphenol talent or dominance (Fig. 3b and Supplementary Fig. 11). While some of these were well characterized polyphenol and aromatic-degrading genera such as *Novosphingobium*^36^, 71% of these genera were undescribed lineages (that is, alphanumeric names), highlighting the power of genome-resolved analyses to provide metabolic contexts for uncultured or poorly characterized lineages.

An additional six genera were both talented and dominant: the Acidobacterial genera *Terracidiphilus* and Palsa-295, the Actinobacterial genus Palsa-504, and the Proteobacterial genera *Acidocella*, FEN-1191 and Bog-1198 (Fig. 3b). To the best of our knowledge, this represents presumably the first description of the polyphenol relevance of these lineages. Underscoring the relevance of these genera in the Stordalen Mire microbiome, MAGs from these six genera contributed between 6–65% of metatranscriptome expression across habitats and depth (Supplementary Fig. 9). Beyond Stordalen Mire, these genera have been reported to be important members of peatland microbiomes^37,38^, for which we provide roles in carbon cycling.

### Polyphenol-active lineages in the Stordalen carbon cycle

Finally, we wanted to reframe how polyphenols integrate into the Stordalen Mire carbon cycle. Our metatranscriptome data refined traditional conceptual models in permafrost^5,25,39^ by illustrating that polyphenols and phenolic acids may be actively used microbial substrates beyond polysaccharides and sugars (Supplementary Fig. 12). Therefore, to begin to place polyphenols in the carbon cycle, we analysed the metatranscriptome expression of MAGs in the six dominant and talented genera.

Illustrating the genus-level conservation of polyphenol talent in these genera, all MAGs expressed transformations at the polymeric, monomeric and phenolic acid levels (Supplementary Fig. 12). MAGs belonging to the Palsa-295, Bog-1198 and Fen-1191 genera in particular expressed genes encoding enzymes for transformations of six different families of monomeric polyphenols (Supplementary Fig. 12 and Data 5). Furthermore, Palsa-295, Palsa-504, Fen-1191 and Bog-1198 members expressed genes for enzymes that decarboxylate benzoic acids, providing a specific example of how these compounds may directly contribute to CO_2_ production (Fig. 1, and Supplementary Fig. 12 and Data 5). Consistent with expanding polyphenol metabolism across broader redox regimes, MAGs from all six genera had active respiratory lifestyles, expressing genes for enzymes in aerobic respiration, iron, nitrogen and/or sulfur reduction (Supplementary Fig. 12 and Data 5). Lastly, we note that in addition to polyphenol-transforming genes, MAGs from all six genera co-expressed genes for polysaccharide depolymerizing enzymes, and all but *Acidocella* MAGs expressed genes for enzymes in sugar degradation pathways. This further emphasizes the need to consider polyphenol transformations in addition to sugars and polysaccharides when assessing peatland carbon cycles. Collectively, these talented and dominant polyphenol-transforming genera illuminate how polyphenol transformations potentially contribute to Stordalen Mire biogeochemistry.

Beyond these six genera, several methane-cycling genera were identified as having capacity for polyphenol transformations, we posit likely for detoxification purposes. Notably, the methanogenic genus Bog-38 (‘*Candidatus* Methanoflorens’), a critical methanogen at Stordalen Mire^5,40^, was a dominant lineage in the bog and fen expressing genes for polymeric polyphenol-active enzymes (Fig. 3 and Supplementary Fig. 11), while *Methanosarcina* and the genus Fen-7 were dominant lineages expressing genes for polymeric polyphenol-active enzymes in the fen. This suggests that some methanogens may have resistance to polyphenols, contradicting the common assertion that methanogens are inhibited by polyphenols, either directly by cellular toxicity or indirectly through the polyphenols binding essential metals needed for enzyme biosynthesis^41^.

Of the five active Bog-38 MAGs, three expressed genes encoding benzoquinone reductase (AA6) and quercetin-2,3-dioxygenase (QueD, Fig. 2 no. 38 and Supplementary Fig. 12). Two of three *Methanosarcina* MAGs expressed genes for peroxidases and AA6, and one of two Fen-7 MAGs expressed genes for AA6. While AA6 has not been studied in methanogens, the enzyme has been suggested to catalyse transformations to detoxify phenolics^34^. Furthermore, QueD uses oxygen to cleave the flavonoid quercetin, releasing carbon monoxide (CO)^42^. This enzyme has also been suggested to serve as a polyphenol detoxification strategy^43^. Intriguingly, the Bog-38 MAGs co-expressed genes for carbon monoxide dehydrogenase (CODH), potentially facilitating the use of the produced CO as an energy or carbon source^44,45^. Beyond these polyphenol dominant genera, MAGs from two other methanogenic genera, Methanobacterium_A and Methanobacterium_B, also co-expressed genes for QueD and CODH (Supplementary Fig. 12). These findings lead us to hypothesize that polyphenol transformations by methanogens could contribute to resistance and may even directly support methanogenesis, although physiological experiments are needed to validate these suppositions.

Of additional relevance to methane cycling in these climate-critical habitats, two facultative methanotrophic genera were polyphenol dominant lineages in the bog: *Methylocella* and *Methylocystis* (Supplementary Figs. 11 and 12, and Data 5). MAGs from both genera exhibited nearly identical gene expression patterns, expressing genes for PO, QueD, the caffeic acid reduction system and 4-hydroxyphenylpyruvate dioxygenase (Hpp) for converting 4-hydroxyphenylpyruvate to homogentisic acid. *Methylocella* also expressed genes for enzymes involved in homogentisic acid degradation, hinting at heterotrophic activity beyond methane oxidation, a supposition that has been supported for other carbon substrates^46,47^. *Methylocystis* further expressed genes for AA6 and vanillyl-alcohol oxidase (AA4). Just as for the methanogens, while the physiological roles of these transformations are unclear and need to be experimentally investigated, the expression of these genes could enable polyphenol degradation, providing growth substrates or polyphenol detoxification. Furthermore, as these lineages are facultative methanotrophs, polyphenol transformation activity could impact CH_4_ release from Stordalen Mire.

## Discussion

**Fig. 4:**
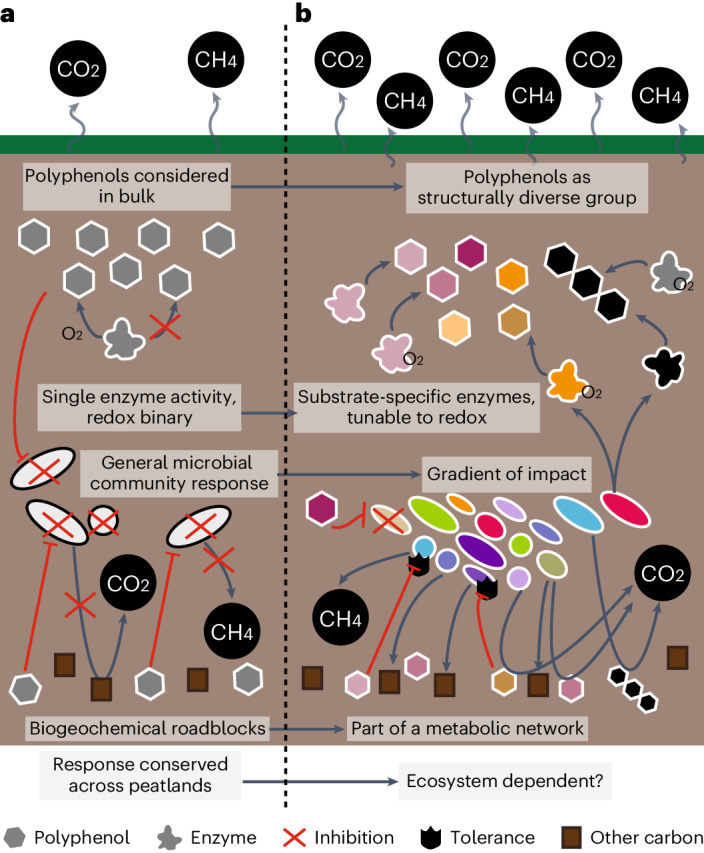
Conceptual reframing of polyphenols in peatland systems. **a**, The enzyme latch model posits that polyphenols can only be degraded by phenol oxidases under oxic conditions. Without oxygen, polyphenols accumulate and generally inhibit the microbial community, subsequently shutting down microbial metabolism, reducing CO_2_ and CH_4_ emissions. **b**, In this study, we propose a polyphenol-cognizant model that accounts for the fact that polyphenols are a diverse group of compounds with diverse metabolic impacts. Microbial communities encode and express multiple substrate-specific enzymes for diverse polyphenols, with strategies under both oxic and anoxic conditions. As a whole, the microbial community exhibits a gradient of responses to polyphenols, ranging from potential inhibition to stimulation. Due to this, polyphenols are integrated into carbon cycling networks, with susceptible organisms, tolerant organisms and stimulated organisms.

Polyphenols have long been cited as controllers of peatland carbon cycles through the enzyme latch mechanism. The studies proposing and supporting this role have historically measured the impact of polyphenols on net outputs of carbon mineralization^7–9,26^. We collected similar data, but also focused on the interplay between polyphenols and the microbiome in situ using multi-omics (Fig. 4). In all, our data refine the enzyme latch theory to be more reflective of modern microbiome science.

We first emphasize that polyphenols are not a bulk entity, but a diverse class of compounds with distinct impacts. We show that microbial communities engage with this chemical diversity, expressing many substrate-specific enzymes for polyphenols. This contrasts with the PO-centric enzyme latch mechanism, which acknowledges just one enzyme type. Furthermore, rather than a redox binary where anoxia inhibits polyphenol decomposition, we found that microbial communities tuned their gene expression strategies to habitat and redox conditions. Therefore, instead of a general antagonistic community response to polyphenols, we illustrated that the microbiome harbours members ranging from polyphenol-inhibited to stimulated. Importantly, we show that rather than shutting down metabolism for the whole microbial community, polyphenols are probably integrated into a larger carbon decomposition network where they serve as substrates, and their decomposition into smaller phenolics could directly fuel carbon dioxide and carbon monoxide production. Rather than relying on a single enzyme, we propose the talented microbiome model where soil microbiomes encode and express a cache of polyphenol metabolisms.

This model raises several questions that should be explored across terrestrial systems. First, the rates of polyphenol removal across the observed transformation pathways remain unknown. Second, the impact of diverse polyphenol degradation pathways for overall carbon storage, the key outcome of the enzyme latch, needs to be examined. Furthermore, the contributions of aerobic and anaerobic fungi to polyphenol degradation should be explored^48,49^, as well as the direct and indirect impacts of polyphenol metabolism on the methane cycle. We highlight CAMPER as a tool to facilitate detection of genes encoding polyphenol-active enzymes in genomic datasets, enabling researchers to begin addressing these questions in their datasets.

As permafrost carbon stores face uncertain fates with changing climates, we must not assume that portions of carbon are microbially unavailable without also considering the vast enzymatic diversity encoded in soil microbiomes. We highlight this work as a starting place for studying polyphenol dynamics in ecosystem carbon cycles in a post-enzyme latch world. Future assessments of peatland and permafrost microbial carbon cycles should consider the myriad ways that polyphenols support microbial metabolism to better understand and to predict ecosystem biogeochemistry.

## Methods

### Phenol oxidase

Here we refer to PO activities instead of a single PO enzyme to best reflect the biochemical understanding of this enzyme group (Supplementary Note 1). We measured PO activity through the l-3,4,-dihydroxy phenylalanine (l-DOPA) assay (see ‘Enzyme assays’). To survey PO gene expression, we used the CAZyme family Auxiliary Activity 1 (AA1) to cover PO activity under Enzyme Commission number 1.10.3.-. Dioxygenases and monooxygenases were identified on their own.

### Field site and sampling

Stordalen Mire is a thawing permafrost peatland in Northern Sweden (68° 22ʹ N, 19° 03ʹ E). The site has three main habitats along a permafrost thaw gradient, with distinct vegetation and thaw status: (1) palsa, overlaying permafrost, well-drained and dominated by woody plants and shrubs; (2) bog, intermediate thaw with variable water table depth characterized by a mixture of woody, herbaceous, sedge and moss plants, dominated by *Sphagnum* mosses and *Eriophorum vaginatum*; and (3) fen, fully thawed and waterlogged, dominated by *Eriophorum angustifolium* and *Carex* spp.

While MAGs were recovered from a longer period of field sampling (see ‘MAG database construction’), all analyses described below were performed on samples collected in July 2016. Triplicate cores were taken from palsa, bog and fen sites (all within a 120 m radius and centred around an autochamber system) in Stordalen Mire using an 11-cm-diameter push corer. Cores were sectioned in the field at three depths: surface (1–5 cm), middle (10–14 cm) and deep (20–24 cm) (Supplementary Fig. 2). Each depth was split into separate samples for microbial and geochemical analyses in the field, immediately upon coring. For microbial analysis, peat from the interior of the core (that is, avoiding the outermost centimetre of core circumference) was added to Lifeguard buffer in the field (QIAGEN) at a ~1:2.5 ratio, shaken vigorously, transported on ice to the research station and stored at −80 °C until shipment on dry ice, then returned to −80 °C storage until analysis. For geochemistry, the samples were stored in 50 ml falcon tubes at −20 °C.

### Geochemical analyses

Before coring, porewater from each core site was collected using a perforated stainless-steel tubing inserted into the peat to the desired depth and extracted with an airtight syringe. No pore water was collected in the dry palsa or above the water table in the bog. Porewater was used to determine pH, and samples were acidified to measure porewater CO_2_ and CH_4_ using a flame-ionization-detector gas chromatograph equipped with a methanizer.

### MAG database construction

To maximize the site-specific genomes available for this study, a database of 13,290 medium and high-quality MAGs was compiled from metagenomes taken from Stordalen Mire peat cores taken in field campaigns from 2010 to 2017 and from a stable isotope probing (SIP) experiment performed on field peat added to labelled plant matter from locally co-occurring species. Notably, SIP metagenomes were used here purely to add site MAGs, not for analyses of that experiment. The methodology of MAG recovery is reported in ref. ^50^. MAG origin is given in Supplementary Data 3.

The 13,290 MAGs (with at least 70% completeness and less than 10% contamination, as determined through CheckM (v.1.0.12)^51^) were dereplicated using galah (v.0.3.0)^52^ with the following parameter set: –precluster-ani 90 –ani 97 –precluster-method finch. This resulted in 1,864 representative MAGs. MAGs were taxonomically annotated using the GTDB-tk (v.2.0)^53^ classify workflow with the GTDB-Tk reference data v.r207. MAGs were annotated using DRAM (v.1.4.0)^54^. MAG taxonomy and checkM statistics are available in Supplementary Data 3. DRAM annotations are available in Zenodo^55^.

### MAG metagenome abundance determination

To determine metagenome abundances of the 97% dereplicated MAG set, we first mapped trimmed metagenome reads to the MAG set using bowtie2 (v.2.4.5)^56^ with the following settings: -D 10 -R 2 -N 1 -L 22 -i S,0,2.50. The output SAM file was converted to a sorted BAM using samtools (v.1.9)^57^ and filtered using the reformat.sh script in the bbtools package using: idfilter=0.95 pairedonly=t primaryonly=t. MAG abundance was inferred from this using coverM (v.0.6.0)^58^ as in ref. ^32^. MAG metagenome abundance is given in Supplementary Data 3.

### RNA extraction

DNA and RNA were co-extracted using the Mobio PowerMax Soil DNA/RNA isolation kit (12966-10) with slight modifications. Briefly, sample vials were removed from the −80 °C freezer and thawed on ice. After thawing, 5–10 g of peat materials (preserved in Lifeguard soil preservation solution, QIAGEN) was added into the bead tubes in the kit, and the nucleic acids were extracted following the manufacturer protocol without the addition of beta-mercaptoethanol at the beginning. Reagents were proportionally increased to maintain the concentration and strength of solutions. An additional ethanol wash of nucleic acids-bound column was performed to further wash out impurities. The resulting nucleic acids were eluted with 5 ml RNase-free deionized water and further concentrated using ethanol precipitation overnight and re-eluted in 100 ml of TE buffer. The resulting nucleic acids were then further processed for DNA and RNA separation and purification. Briefly, the extracted nucleic acids were aliquoted into two 2 ml tubes at a ratio of 1:2. RNase treatment and DNase treatment (Roche) were performed following manufacturer instructions on the recovery of DNA and RNA. After the treatments, DNA and RNA were further purified by phenol:chloroform purification to remove the enzymes and impurities. DNA and RNA were then ethanol precipitated and the pellets were eluted in TE buffer. Final purified DNA and RNA were quantified with Qubit 3.0. Quality of extracted RNA was also examined using TapeStation (Agilent) analysis at the Genome Shared Resources at the Ohio State University. Extracts were stored at −80 °C until downstream sequencing analysis.

### Metatranscriptome analysis

Metatranscriptome libraries were prepared for the 27 July 2016 samples. Using 10 ng of RNA as input, ribosomal RNA was depleted using the QIAseq FastSelect −5S/16S/23S (QIAGEN, 335921) kit following the kit protocol with the following modifications: addition of probes for plant and yeast, and using one-third of the probe volumes. Then, the TruSeq Stranded mRNA Library Preparation kit (Illumina, 20020595) was used to prepare the sequencing library. Libraries were sequenced on an Illumina NovaSeq 6000 system (v.1.5 chemistry, S4 flow cell, 2 × 150 bp) at the Genomics Shared Resource Facility at the University of Colorado Anschutz Medical Campus.

Raw metatranscriptome reads were quality trimmed and adapters removed using bbduk^59^ with the following flags: k = 23 mink=11 hdist=1 qtrim=rl trimq=20 minlength=75. Reads were filtered using rqcfilter2 (ref. ^59^) with the following flags: jni=t rna=t trimfragadapter=t qtrim=r trimq=0 maxns=1 maq=10 minlen=51 mlf=0.33 phix=t removeribo=t removehuman=t removedog=t removecat=t removemouse=t khist=t removemicrobes=t mtst=t sketch kapa=t clumpify=t tmpdir=null barcodefilter=f trimpolyg=5. Filtered and trimmed reads were mapped against the database of 97% dereplicated MAGs using bowtie2 (ref. ^56^) with the following flags: -D 10 -R 2 -N 1 -L 22 -i S,0,2.50. The output SAM file was converted to BAM using samtools^57^ and filtered using the reformat.sh script in the bbtools package using: idfilter=0.95 pairedonly=t primaryonly=t. Mapped reads were counted using htseq-count (v.0.13.5)^60^ with the following flags: -a 0 -t CDS -i ID–stranded=reverse. Read counts were filtered to remove counts <5 and were converted to geTMM^61^ in R. Scripts for processing metatranscriptome count data are provided in GitHub (see Data Availability). Metatranscriptome data are given in Supplementary Data 3 and gene-level mapping data are deposited in Zenodo^62^.

### CAMPER polyphenol annotation construction and curation

CAMPER uses a mixture of database-derived and custom annotation searches. Database-derived searches include 272 Hidden-Markov Models (HMMs) from KofamScan^63^, using the provided bitscore cut-offs for each HMM, and 5 HMMs from dbCAN2 (ref. ^64^) using the suggested bitscore cut-off (bitscore=105). For the custom annotation searches, we constructed 8 HMMs and 33 Basic Local Alignment Search Tool (BLAST) searches (Supplementary Table 1). HMMs were created in cases where sufficient biochemically characterized sequences existed (defined as sequences from at least 3 microbial genera, see Supplementary Table 1). We refer to these biochemically characterized sequences as ‘seed sequences’. An overview of the HMM construction workflow is given in Supplementary Fig. 13. Briefly, for each gene type, we identified ‘decoy sequences’ that represented related but functionally distinct sequences to the gene of interest. To create the HMM profiles, we used BLASTP to compare the seed sequences and the decoy sequences against the UniProt90 (ref. ^65^) database and pulled the top 200 hits for each gene (referred to here as seed homologues and decoy homologues, respectively). Using the seed and decoy homologue sequences, seed sequences and decoy sequences, we created a sequence alignment using MAFFT (v.7.055b)^66^ with ‘-auto’ flag, and the alignment was trimmed using trimal (v.1.4.rev22) -gappyout^67^. This trimmed alignment was fed to IQTree^68^ (v.1.6.8) with the following flags: -alrt 1000 -bb 1000 -m MFP -nt AUTO -ntmax 10. The ‘.tree’ file was visualized in iTOL^69^ and rooted on the clade containing all decoy sequences. Then, we identified the clade that contained all seed sequences and pulled the seed homologue sequences from this clade. Importantly, we removed the seed sequences from this set to serve as a hold-out dataset when evaluating HMM quality. Using the seed homologue sequences, we constructed HMMs in graftM^70^ (v.0.13.0) using the graftM create command, and searched them against the seed sequences, seed homologue sequences, decoy homologues and decoy sequences using the graftM graft command. Seed sequences, trimmed alignments, tree files and HMM seed alignments can be found on the CAMPER GitHub page (https://github.com/WrightonLabCSU/CAMPER/).

We curated score cut-offs to balance annotation precision and recall. We made two score cut-offs: the ‘A’ score was the lowest bitscore assigned to a seed sequence. As these sequences were not used in HMM construction, this score was designed to increase annotation precision (Supplementary Fig. 14). The ‘B’ score was curated from a combination of tree placement and score distribution. We provided a B-score to increase annotation recall (Supplementary Fig. 14), recognizing the potential for organismal bias in the seed sequences. If there was not a clear ‘B’ score cut-off, we opted to only provide an ‘A’ score. Importantly, we ensured that ‘A’ and ‘B’ score thresholds were greater than bitscores obtained from searching the HMM against decoy sequences and homologues to minimize false positive annotations. BLAST searches were used for genes lacking sufficient numbers of and/or diversity in seed sequences (Supplementary Table 1). We used BLAST bitscores of 200 and 120 as the ‘A’ and ‘B’ score thresholds, respectively.

We validated CAMPER on a set of 5 experimentally characterized isolate genomes (GCA_004345805, GCA_000469345, GCA_000409755, GCA_000478885, GCA_003725955)^71^. We show that CAMPER genome annotations match the experimental phenotypes for these isolates across three polyphenol metabolic pathways (Supplementary Fig. 14 and Data 2). These metabolisms were covered by 7 HMMs and 1 BLAST search. Owing to the lack of experimental data paired to genome sequences, validation of the remaining 1 HMM and 32 BLAST searches was not possible. We note that similar to all homology-based gene annotators, CAMPER provides predicted annotations. Users should carry out additional curation, when possible; for example, curating pathway completion or checking gene organization (known gene organization is noted in the CAMPER output ‘note’ column).

CAMPER was used to annotate the 97% dereplicated MAG set, and all A and B-score annotations were noted. These annotations were combined with DRAM annotation outputs to evaluate the 100 transformation pathways shown in Supplementary Data 2. MAGs were said to encode a transformation pathway if they possessed >50% of the genes in the pathway. In the genome-resolved metatranscriptome data, MAGs were said to express the genes of a pathway if they encoded >50% of the genes in the pathway, and at least 1 gene recruited metatranscriptome reads (read counts >5).

We defined two metrics of polyphenol importance at the genus level: talent and dominance. To assess this, we aggregated the expression of MAGs within each genus. Polyphenol talent was defined at the genus level as expression of genes involved in at least 15 unique transformation pathways in each habitat (across depths). This was chosen as 15 pathways corresponded to a *P* value of 0.05 in the overall distribution (Supplementary Fig. 10). Polyphenol dominance was defined at the genus level as contributing an average of at least 10% of polymer, monomer or phenol active expression in a habitat (per depth). This was chosen as 10% corresponded to a *P* value of 0.05 in the overall distribution (Supplementary Fig. 10).

### MAG phylogenetic tree construction

Phylogenetic trees of the MAGs were inferred using the GTDB de_novo_wf workflow for both bacterial and archaeal MAGs, using p__Patescibacteria and p__Micrarchaeota as outgroups, respectively. Phylogenetic trees were visualized using the ggtreeEXTRA^72,73^ R package.

### Methanogen and methanotroph annotation curation

To confirm the identity of environmental methanogen 1,4-benzoquinone reductase (AA6) and quercetin-2,3-dioxygenase (K06911) sequences, all were submitted to Phyre2 (ref. ^74^) for structural modelling to confirm proposed functions. Following modelling, further validation was performed via alignment of methanogen sequences to biochemically validated or crystallized proteins using Clustal^75^ to look for conservation of catalytic residues. The peroxidase sequences were also analysed via the Peroxiscan tool of RedOxiBase^76^ to confirm family placement, and both the peroxidases and the quercetin-2,3-dioxygenases were analysed using SignalP^77^ and PSORTb^78^ to confirm their cytoplasmic status. Details on this curation can be found in Supplementary Data 5.

### Enzyme assays

The activities of the five hydrolytic enzymes β-d-glucosidase, β-d-xylosidase, *N*-acetyl-β-d-glucosaminidase, arylsulphatase and phosphatase were assessed using fluorometric enzyme assays following methods adapted from refs. ^79,80^. Briefly, a soil slurry was prepared for each sample by blending 1 g of peat with 125 ml of sodium acetate buffer (50 mM, pH 6.2). The soil slurry was then transferred to a 96-well flat-bottom black microplate which included a buffer-only control column, as well as controls containing only soil and standard. Subsequently, 4-methylumbelliferyl (MUB) standard solution and fluorescently linked enzyme substrates were added to the respective wells, and the plates were incubated at 25 °C for 45 min for β-d-glucosidase, *N*-acetyl-β-d-glucosaminidase, arylsulphatase and phosphatase, and for 30 min for β-d-xylosidase. Incubation times and substrate concentrations were chosen on the basis of a *V*-max test performed to capture peak enzyme activity. Fluorescence was read in a BioTek Synergy HT microplate reader at a wavelength of 460 nm emission and 360 nm excitation. Final enzyme activity was reported as μmol activity g^−1^ dry soil h^−1^.

The oxidative enzyme activity of phenol oxidase was assessed using a colorimetric enzyme assay^80^. A soil slurry was prepared for each sample by blending 1 g of peat with 125 ml of sodium acetate buffer (50 mM, pH 6.2), and slurries were transferred to a 96-deep-well plate. A blank column containing only buffer was included in the plate, as well as controls containing only buffer and substrate. l-DOPA was chosen as the substrate for measuring polyphenol oxidase activity. After adding 25 mM l-DOPA substrate, plates were incubated at 25 °C for 24 h. Following incubation, the supernatant was transferred to a 96-well flat-bottom clear microplate and absorbance was read in a BioTek Synergy HT microplate reader at 460 nm. Final activity was reported as µmol activity g^−1^ dry soil h^−1^. Enzyme assay data are provided in Supplementary Data 1, and are available in Zenodo^81^.

### Folin–Ciocalteu assay

To determine total phenolics, we used the Folin–Ciocalteu assay on the water extracts of the soil samples. Extracts were prepared from all the samples, centrifuged to remove soil and stored at −80 °C until analysis. On the day of analysis, each extract was thawed and centrifuged briefly (10,000 × *g*) to remove insoluble material, and 25 μl aliquots were transferred to 96-well plates. The methyl-gallate standard (0.2 mg ml^−1^) was dispensed into the wells to obtain a series of samples containing 0-4.25 μg methyl gallate. On each plate, both samples and standards were run in triplicate. Each extract was analysed on two different days. After adding 50 μl of Folin reagent (Sigma), 20 μl of 20% (m/v) Na_2_CO_3_ and enough water to bring the volume of each well to 95 μl, the plate was briefly mixed and then incubated in the dark for 20 min. The samples were then read at 750 nm (Biotek Synergy LX), a standard curve was generated and the total phenolic content of each sample established by interpolation. Folin–Ciocalteu assay data are provided in Supplementary Data 1.

### Organic matter metabolite extraction

Water-soluble metabolites were extracted from peat by adding 7 ml of autoclaved milliQ water to 1 g of wet peat in a sterile 15 ml centrifuge tube. Tubes were vortexed twice for 30 s, and then the peat–water mixture was sonicated for 2 h at 22 °C. Samples were then centrifuged to separate the supernatant (6 ml) which served as the water extract. Samples were kept at −80 °C until analysis.

### LC–MS

Samples were prepared for LC–MS/MS metabolomics as previously described^50^. Briefly, water-extracted metabolites were thawed at room temperature and centrifuged again to remove any particles that potentially formed after thawing. Next, each sample was split into two 2 ml glass tube vials (1 ml each), one for hydrophilic interaction liquid chromatography (HILIC) and the other for reverse-phase liquid chromatography (RPLC). Samples in both vials were dried down completely on a Vacufuge plus (Eppendorf) and resuspended in a solution of 50% acetonitrile and 50% water for HILIC and a solution of 80% water and 20% HPLC-grade methanol for RPLC.

A Thermo Scientific Vanquish Duo ultra-high performance liquid chromatography system (UHPLC) was used for the liquid chromatography step. Extracts were separated using a Waters ACQUITY HSS T3 C18 column for RP separation and a Waters ACQUITY BEH amide column for HILIC separation.

Samples were injected in a 1 μl volume on the column and eluted as follows: for RPLC, the gradient went from 99% mobile phase A (0.1% formic acid in H_2_O) to 95% mobile phase B (0.1% formic acid in methanol) over 16 min. For HILIC, the gradient went from 99% mobile phase A (0.1% formic acid, 10 mM ammonium acetate, 90% acetonitrile, 10% H_2_O) to 95% mobile phase B (0.1% formic acid, 10 mM ammonium acetate, 50% acetonitrile, 50% H_2_O). Both columns were run at 45 °C at a flow rate of 300 μl min^−1^.

A Thermo Scientific Orbitrap Exploris 480 was used for spectral data collection at a spray voltage of 3,500 V for positive mode (for RPLC) and 2,500 V for negative mode (for HILIC) using the heated-electrospray ionization (H-ESI) source. The ion transfer tube and vaporizer temperature were both 350 °C. Compounds were fragmented using data-dependent MS/MS with HCD collision energies of 20, 40 and 80.

The Compound Discoverer 3.3.2.31 software (Thermo Fisher) was used to analyse the data using the untargeted metabolomics workflow. Briefly, the spectra were first aligned, followed by a peak-picking step. Putative elemental compositions of unknown compounds were predicted using the exact mass, isotopic pattern, fine isotopic pattern and MS/MS data using the built-in HighChem Fragmentation Library of reference fragmentation mechanisms. Metabolite annotation was performed using an in-house database built using 1,200 reference standards, spectral libraries and compound databases. First, fragmentation scans, retention times and ion masses of unknown compounds were compared with those in the in-house database. Second, fragmentation scans (MS2) searches were performed in mzCloud, which is a curated database of MSn spectra containing more than 9 million spectra and 20,000 compounds. Third, predicted compositions were obtained on the basis of mass error, matched isotopes, missing number of matched fragments, spectral similarity score (calculated by matching theoretical and measured isotope patterns), matched intensity percentage of the theoretical pattern, the relevant portion of MS and the MS/MS scan. The mass tolerance used for estimating predicted composition was 5 ppm. Finally, annotation was complemented by searching MS1 scans in different online databases with ChemSpider (using either the exact mass or the predicted formula). The compounds’ level of annotation was assigned according to the Metabolomics Standards Initiative as follows^82^: Level 1: compounds with exact match to a standard reference compound in our in-house library; Level 2: compounds with full match to online spectral databases using the mzCloud database (based on MS2 spectra matching) and ChemSpider (using mass and molecular formula generated through the Predicted Compositions node). Annotations were manually inspected for validation, identification of isomers and removal of in-source fragments.

To enhance annotation coverage and add compound classes, SIRIUS, CSI:FingerID and CANOPUS were used^83^. Compound chemical taxonomy based on chemical structure was assigned using ClassyFire^84^. For annotated polyphenols, chemical taxonomy was assigned by hand using the following rules: phenolic acids contain a single aromatic ring with only hydroxyl and ether groups attached; benzoic acids contain a single aromatic ring with a carboxylic acid or ester attached in the alpha position of the carbonyl; flavonoids are 15-carbon members of a heteroatom connected to an aromatic ring; lignans contain two aromatic rings bound by a 4-carbon chain; stilbenes are two aromatic rings separated by two carbons with a double bond between them; and others are aromatic compounds that do not fit into the above categories. LC–MS/MS data are given in Supplementary Data 4.

### Fourier-transform ion cyclotron resonance MS analysis

Water extracts (3 ml) were first purified using solid-phase extraction to remove contaminants (salts) according to ref. ^85^. Briefly, water extracts were acidified to pH 2 using 1 M HCl. Then, extracts were filtered through a 3 ml Bond Elut PPE cartridge (Agilent) that was previously activated using methanol. Cartridges were washed with 3 ml of a 0.01 M HCl solution five times, then dried using filtered air. Finally, extracts were eluted using 1.5 ml of methanol and stored at −80 °C until use.

Purified extracts were analysed by direct injection using a 12 Tesla Bruker Fourier-transform ion cyclotron resonance (FT-ICR) mass spectrometer located at the Pacific Northwest National Laboratory. Negative charged molecular ions were generated using a Bruker ESI source. Instrument stability was optimized using a Suwannee River Fulvic Acid standard (SRFA), obtained from the International Humic Substance Society. Potential carry-over between samples was monitored by injecting HPLC-grade methanol. The instrument was flushed between samples using a combination of milliQ water and methanol. To account for variations in carbon concentrations in different samples, the ion accumulation time was varied between 0.03 and 0.05 s. A total of 144 individual scans per sample were collected, averaged and calibrated using an organic matter homologous series separated by 14 Da (CH2). Mass accuracy was <1 ppm for single charged ions measured across an *m*/*z* range of 100–1,200 *m*/*z*, the mass resolution was ~240 K at 341 *m*/*z* and the transient was 0.8 s. Raw spectra collected per sample were transformed into a list of *m*/*z* values using the FT-MS peak picker module within the BrukerDaltonik (v.4.2) software using a signal to noise ratio of 7 and absolute intensity threshold of 100 (default). Formularity^86^ software was used to assign putative chemical formulae following ref. ^87^. FT-ICRMS data are available in Supplementary Data 4 and in Zenodo^88^.

### Refinement of tannin and lignin Van Krevlen classes

Van Krevlen analyses assign molecular formulae to classes using H:C and O:C ratios^89^. Two classical regions are the lignin-like and tannin-like regions; however, these regions mask a huge amount of complexity (for example, condensed tannins vs hydrolysable tannins). Using an approach similar to that in ref. ^90^, we sought to refine these classifications using known formulae from over 60 characterized natural substrates (Supplementary Data 6 and Fig. 15)^91–102^. We determined the following H:C and O:C ratios for hydrolysable tannins (HT), condensed tannins (CT) and lignin (lig):

Lignin: 0.3 < O:C < 0.48, 1.08 < H:C < 1.28

Hydrolysable tannin: 0.6 < O:C < 0.7, 0.58 < H:C < 0.89

Condensed tannin: 0.4 < O:C < 0.5, 0.74 < H:C < 0.88

We used the CT, HT and lig class boundaries to classify features. To determine the relative abundance of these classes, we summed the abundance of features for each class within each sample. We then averaged this across replicates for each class within a habitat and depth. To determine ‘percent polyphenol’ (Fig. 1), we summed the percent CT, percent HT and percent lig.

### Statistics

All data analyses and visualization were done in R (v.4.2.1)^103^ with the following packages: stats, cowplot (v.1.1.1)^104^, ggplot2 (v.3.3.6)^105^, ggtreeEXTRA (v.1.6.0)^72^, tidyr (v.1.2.0)^106^, dplyr (v.1.0.9)^107^, readxl (v.1.4.0)^108^, pheatmap (v.1.0.12)^109^, RColorBrewer (v.1.1-3)^110^, edgeR (v.3.16)^111^, stringr (v.1.4.0)^112^ and writexl (v.1.4.0)^113^. For statistical analyses of enzyme latch data types, we used Mann–Whitney test and Pearson correlations in R, and *P* values were adjusted within groups of comparisons using Benjamini–Hochberg adjustment.

### Reporting summary

Further information on research design is available in the Nature Portfolio Reporting Summary linked to this article.

### Supplementary information

Supplementary Information Author Consortia Members, Supplementary Note 1, Figs. 1–13, Table 1 and References.

Reporting Summary

Supplementary Data 1 Sample metadata and data underlying Fig. 1.

Supplementary Data 2 MAG polyphenol genome potential and metatranscriptome expression.

Supplementary Data 3 Metagenome and metatranscriptome information.

Supplementary Data 4 LC–MS/MS and FT-ICRMS data.

Supplementary Data 5 Metabolism curation of the polyphenol talented and dominant genera.

Supplementary Data 6 Data used to refine classifications of polyphenol-like classes.

## Data Availability

The metagenomes, metatranscriptomes and most metagenome-assembled genomes used in this paper are available at NCBI under BioProjectID PRJNA386568. See Supplementary Data 3 for individual BioSample numbers. All MAGs are available via Zenodo at 10.5281/zenodo.7596016 (ref. ^114^). All raw and processed data are available in the following Zenodo archives: MAG annotations at 10.5281/zenodo.7587534 (ref. ^55^), metatranscriptome mapping at 10.5281/zenodo.7591900 (ref. ^62^), FT-ICRMS at 10.5281/zenodo.7519321 (ref. ^88^) and enzyme assays at 10.5281/zenodo.7519395 (ref. ^81^). The following publicly available databases were used in this study: UniProt90 (https://www.uniprot.org/help/uniref), RedOxiBase (https://peroxibase.toulouse.inra.fr/), SignalP (https://services.healthtech.dtu.dk/services/SignalP-6.0/), pSortB (https://www.psort.org/psortb/) and KOfam (https://www.genome.jp/ftp/db/kofam/).

## Code availability

All scripts involved with data generation, processing, curation and visualization are available on GitHub (https://github.com/WrightonLabCSU/stordalen_polyphenols_paper). The CAMPER annotation tool is available on GitHub (https://github.com/WrightonLabCSU/CAMPER).
