# Supplementary material for: Microbial polyphenol metabolism is part of the thawing permafrost carbon cycle
Source: Nat Microbiol. 2024 May 28;9(6):1454–66. doi: 10.1038/s41564-024-01691-0 (PMC11153144; doi:10.1038/s41564-024-01691-0)
Supplement: Supplementary file 1 — Author Consortia Members, Supplementary Note 1, Figs. 1–13, Table 1 and References. [file 41564_2024_1691_MOESM1_ESM.pdf]

nature microbiology

Supplementary information https://doi.org/10.1038/s41564-024-01691-0

Microbial polyphenol metabolism is part of the thawing permafrost carbon cycle

In the format provided by the authors and unedited

The EMERGE 2016 Field and Analysis Team Members and Affiliations
Hanna Axén[1], Moira Hough[2], Nicole Irwin-Raab[3], Joachim Jansen[4], Yueh-Fen Li[3], Ruth K. Varner[5]

1. Department of Ecology and Environmental Science, Umeå University, Umeå, Sweden
2. College of Forest Resources and Environmental Science, Michigan Technological University, Houghton, MI, USA, 49931
3. Department of Microbiology, The Ohio State University, Columbus, OH, USA, 43210
4. Department of Ecology and Genetics, Uppsala University, Uppsala, Sweden
5. Department of Earth Sciences and Earth Systems Research Center, University of New Hampshire, Durham, NH, USA 03824

The EMERGE Institute Coordinators and Affiliations
Sarah C. Bagby[1], Benjamin Bolduc[2], Eoin L. Brodie[3,4], Jeffrey P. Chanton[5], Patrick Crill[6], Jessica G. Ernakovich[7], Maria Florencia Fahnestock[7], Regis Ferriere[8], Suzanne B. Hodgkins[2], Michael Ibba[9], Virginia I. Rich[2], Scott R. Saleska[8], Matthew B. Sullivan[2], Malak M. Tfaily[10], Gene W. Tyson[11], Ruth K. Varner[12], Rachel M. Wilson[5], Ben J. Woodcroft[11], Kelly C. Wrighton[13], Ahmed A. Zayed[2]

1. Department of Biology, Case Western Reserve University, Cleveland, OH, USA, 44106
2. Department of Microbiology, The Ohio State University, Columbus, OH, USA, 43210
3. Earth and Environmental Sciences, Lawrence Berkeley National Laboratory, Berkeley, CA, USA
4. Department of Environmental Science, Policy, and Management, University of California Berkeley, Berkeley, CA, USA
5. Earth Ocean and Atmospheric Sciences, Florida State University, Tallahassee, FL, USA
6. Department of Geological Sciences and Bolin Centre for Climate Research, Stockholm University, Stockholm, Sweden
7. Department of Natural Resources and the Environment, University of New Hampshire, Durham, NH, USA 03824
8. Department of Ecology and Evolutionary Biology, University of Arizona, Tucson, AZ, 85721, USA
9. Schmid College of Science and Technology, Chapman University, Orange, CA, USA
10. Department of Environmental Science; University of Arizona, Tucson, AZ, 85721, USA
11. Centre for Microbiome Research, School of Biomedical Sciences, Queensland University of Technology (QUT), Translational Research Institute, Woolloongabba, QLD, Australia
12. Department of Earth Sciences and Earth Systems Research Center, University of New Hampshire, Durham, NH, USA 03824

Supplementary Note 1

Phenol oxidases (POs) are not a singular enzyme, but rather a group of biochemically distinct enzymes. POs have the characteristic ability to oxidize phenolic compounds using oxygen as an electron acceptor. PO activity has historically been assayed by colorimetrically tracking oxidation of a phenolic compound, most often L-dihydroxyphenylalanine (L-DOPA), though it is important to note that this method is known to be susceptible to mineral interference and secondary oxidation reactions that obscure true activity measurements[1]. This method also does not inform on the identity of enzymes acting upon L-DOPA. PO activity spans enzymes including: tyrosinase and catechol oxidase (EC:1.10.3.1); laccases and polyphenol oxidases (EC:1.10.3.2); dioxygenases (EC:1.13.11); monooxygenases (EC:1.14.18)[1]. However, traditional enzyme latch theory papers have not fully recognized the imprecise nature of PO, instead referring to PO as “a single enzyme”[2] or “the enzyme responsible for the breakdown of phenolic compounds”[3]. In the most nuanced description of PO, extracellular and intracellular phenol oxidase were distinguished[4]. In summary, PO activity can be assigned to numerous enzymes, though this has not always been clearly stated in the enzyme latch theories.

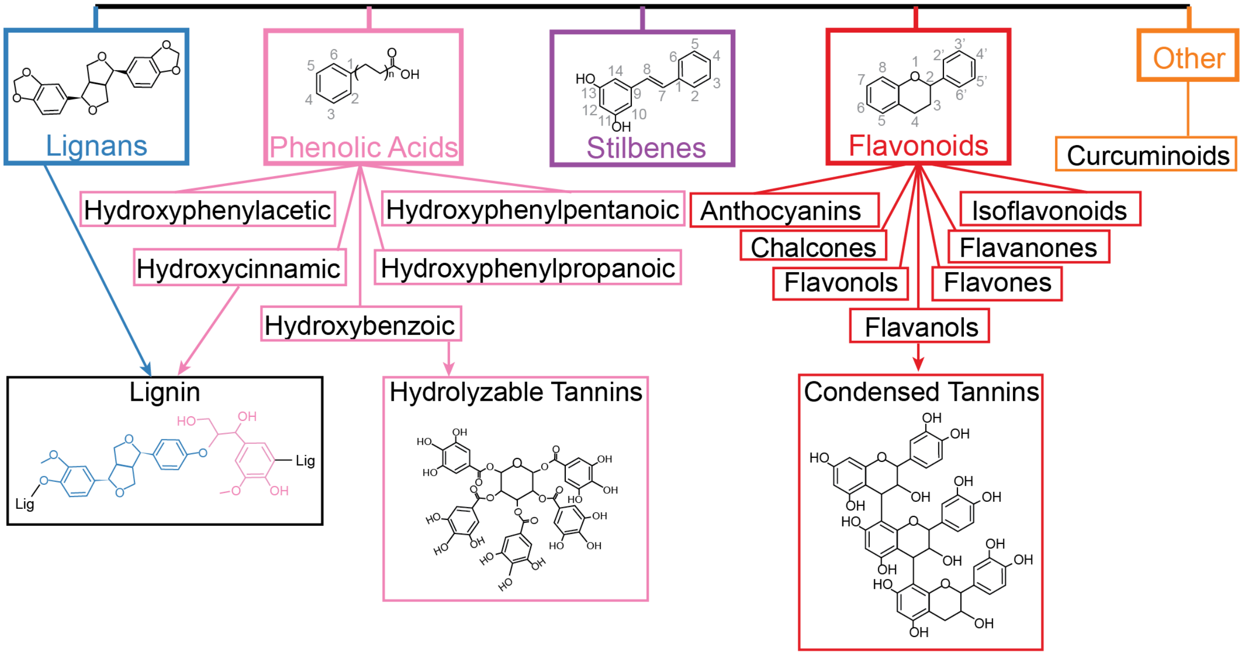

Supplementary Figure 1. The structural diversity of polyphenols. The five major families of polyphenols are shown, followed by their subfamilies. Subfamilies listed under “Other” are not exhaustive. Grey numbers indicate ring positions. These structures can be found in polymerized forms, lignin, hydrolyzable tannins, or condensed tannins, which are shown in boxes.

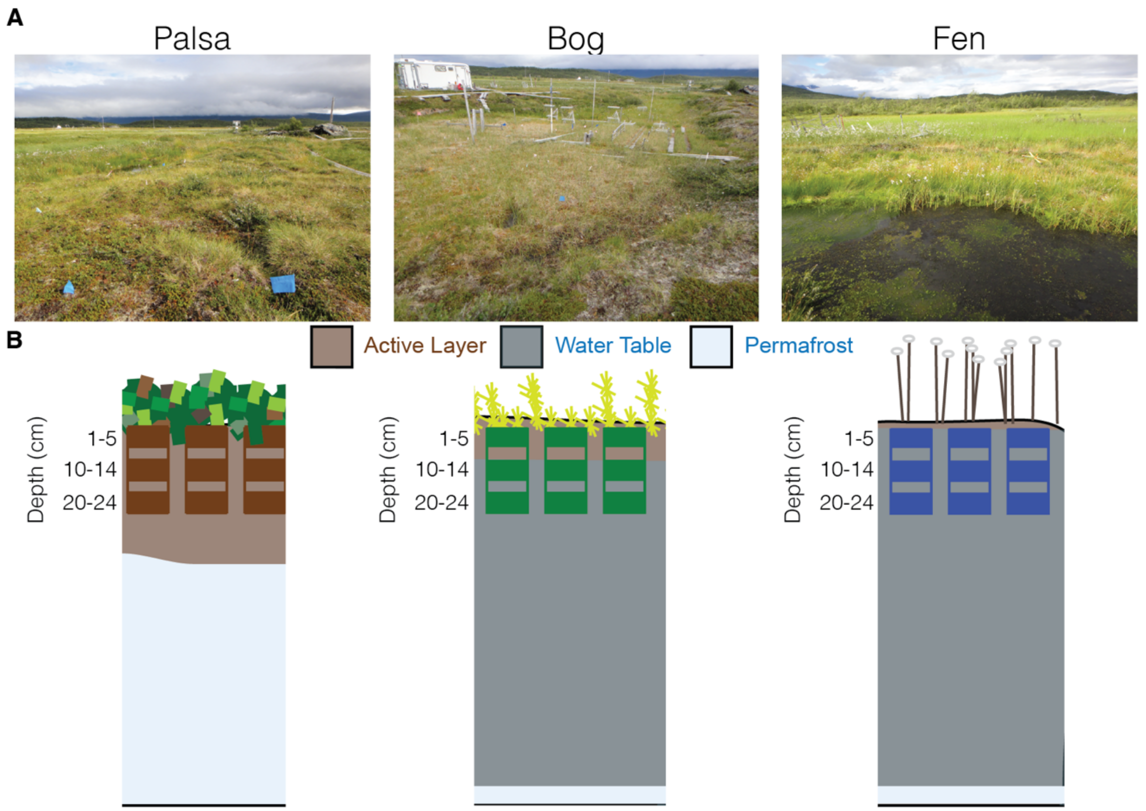

Supplementary Figure 2. Overview of Stordalen Mire habitats and sampling. (**A**) Images of the palsa, bog, and fen sites sampled in July 2016. (**B**) Schematics representing core sampling at each site. Triplicate cores were taken within sites pictured in (**A**) and subsampled, with three depths the focus here: surface (1-5cm), middle (10-14 cm), and deep (20-24 cm) below the surface. The relative position of cores to permafrost layer and water table are depicted by color shadings indicated in the legend (**Supplementary Data 1**).

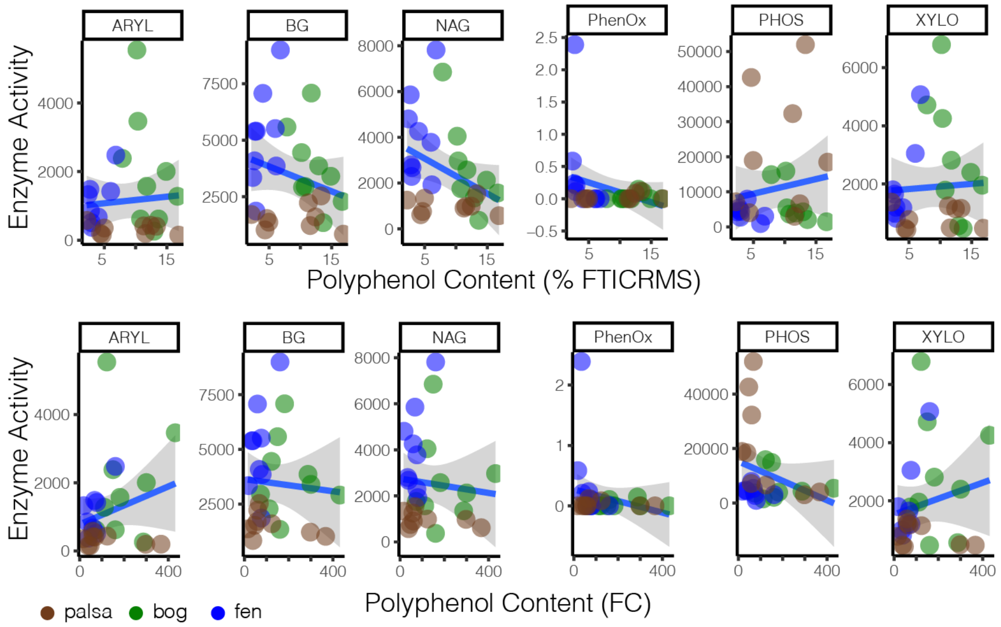

Supplementary Figure 3. **Relationships between different measures of polyphenol content and measured hydrolase enzyme activities.** Polyphenol content from FT-ICRMS (top row) and Folin-Ciocalteu assay (bottom row) are shown. Arylsulfatase (ARYL), beta-glucosidase (BG), N-acetylglucosaminidase (NAG), polyphenol oxidase (PhenOX), phosphomonoesterase (PHOS), and xylosidase (XYLO). The Benjamini-Hochberg adjusted p-values of Pearson's correlations are greater than 0.05 for all correlations (see **Supplementary Data 1** for p-values). Data points are colored by the habitat: palsa (brown), bog (green), and fen (blue). The blue line represents the linear trendline across the data points, and the grey shaded region corresponds to the 95% confidence interval.

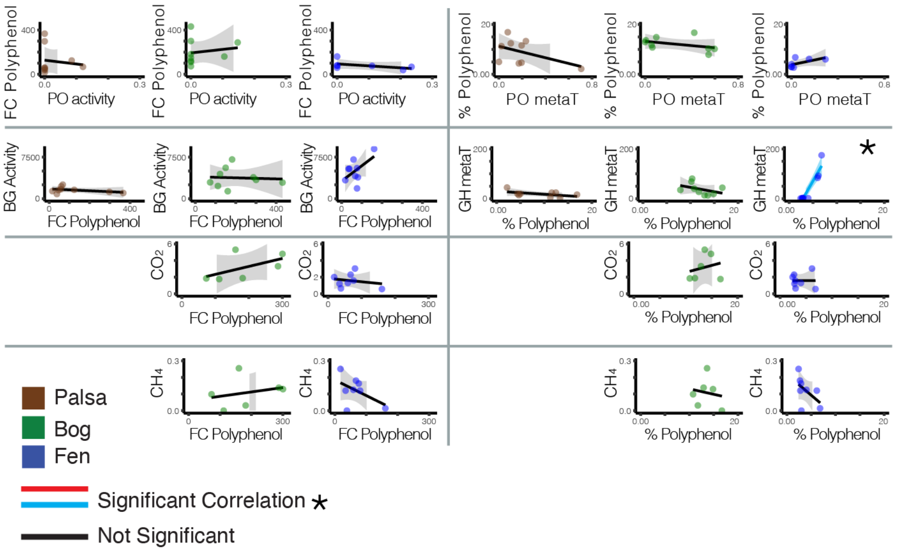

Supplementary Figure 4. Enzyme latch relationships within Stordalen Mire habitats. Significant Pearson correlations (Benjamini-Hochberg adjusted $p<0.05$) are colored blue and red for positive and negative correlations, respectively. Phenol Oxidase activity is given by Phenol Oxidase assay (PO activity, nmol activity/g/hr) and metatranscriptome expression of polyphenol oxidase (PO metaT, geTMM). Polyphenol content is given by Folin-Ciocalteu phenolics (FC-Polyphenol, mg methyl gallate equivalents/dry g soil) and polyphenol-like compounds identified in FTICRMS (% Polyphenol). Extracellular hydrolase enzymes (EHE) are given by Beta-glucosidase activity (BG Activity, nmol activity/g soil/hr) and glycoside hydrolase expression in metatranscriptome (GH metaT, geTMM). Porewater carbon dioxide (CO_2) and methane (CH_4) is given in concentrations (mM). The black line represents the linear trendline across the data points, and the grey shaded region corresponds to the 95% confidence interval. Individual correlation values and sample sizes are given in **Supplementary Data 1**.

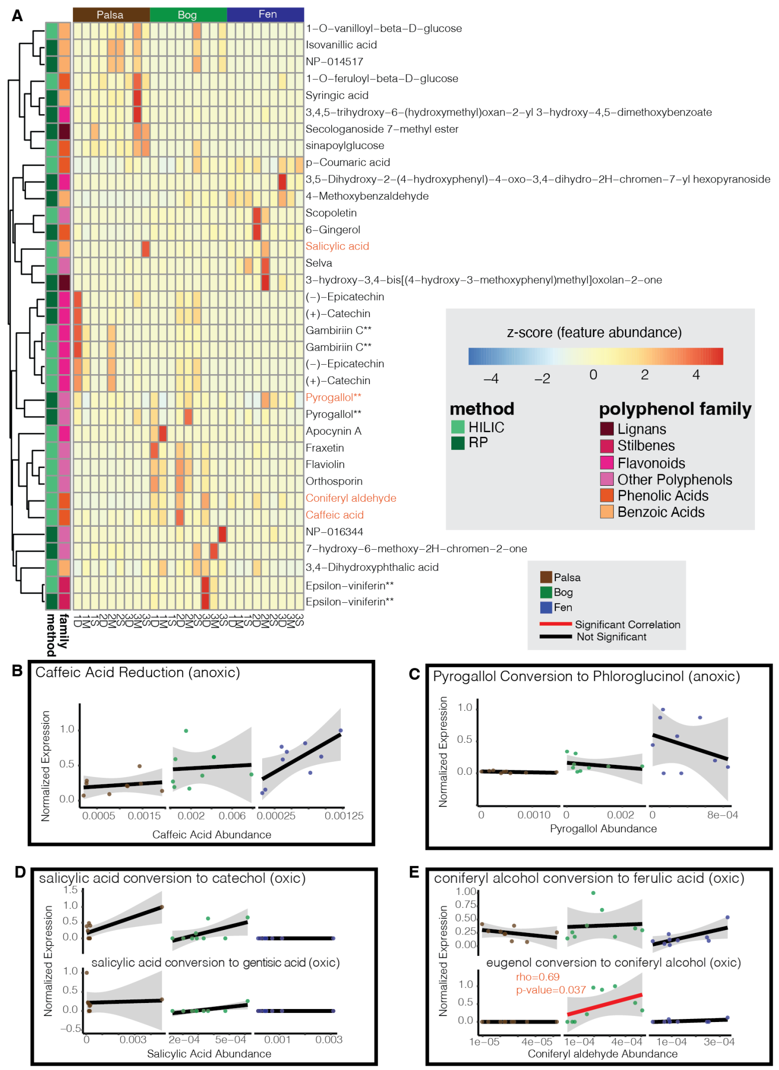

Supplementary Figure 5. Polyphenol metabolites detected in Stordalen Mire. (A) Heatmap of the relative abundance of 35 LC-MS/MS identified polyphenols. Heatmap color represents the z-score (across a row) of the relative abundance of each feature. At left, the heatmap is annotated with the LC-MS/MS method by which the feature was identified: reverse phase with positive ion mode (RP, dark green) or Hydrophobic Interaction Liquid Chromatography with negative ion mode (HILIC, light green). The polyphenol family was curated manually when possible, otherwise using ClassiFyre or CANOPUS (**Supplementary Data 4**). Compound names in red are plotted in B-D. Names with (**) indicate features that are likely isomers. Heatmap columns represent samples, labelled for site at top, and core number and depth at bottom (S= surface, M= Middle, D= Deep). (B-D) Relationship between metabolite abundance and metatranscriptome expression for metabolites that were substrates (caffeic acid, pyrogallol, salicylic acid), products (caffeic acid), or intermediates (coniferyl aldehyde) in polyphenol transformation pathways. The black line represents the linear trendline across the data points, and the grey shaded region corresponds to the 95% confidence interval. Significant Spearman's correlations are given by red lines, with rho and p-value noted.

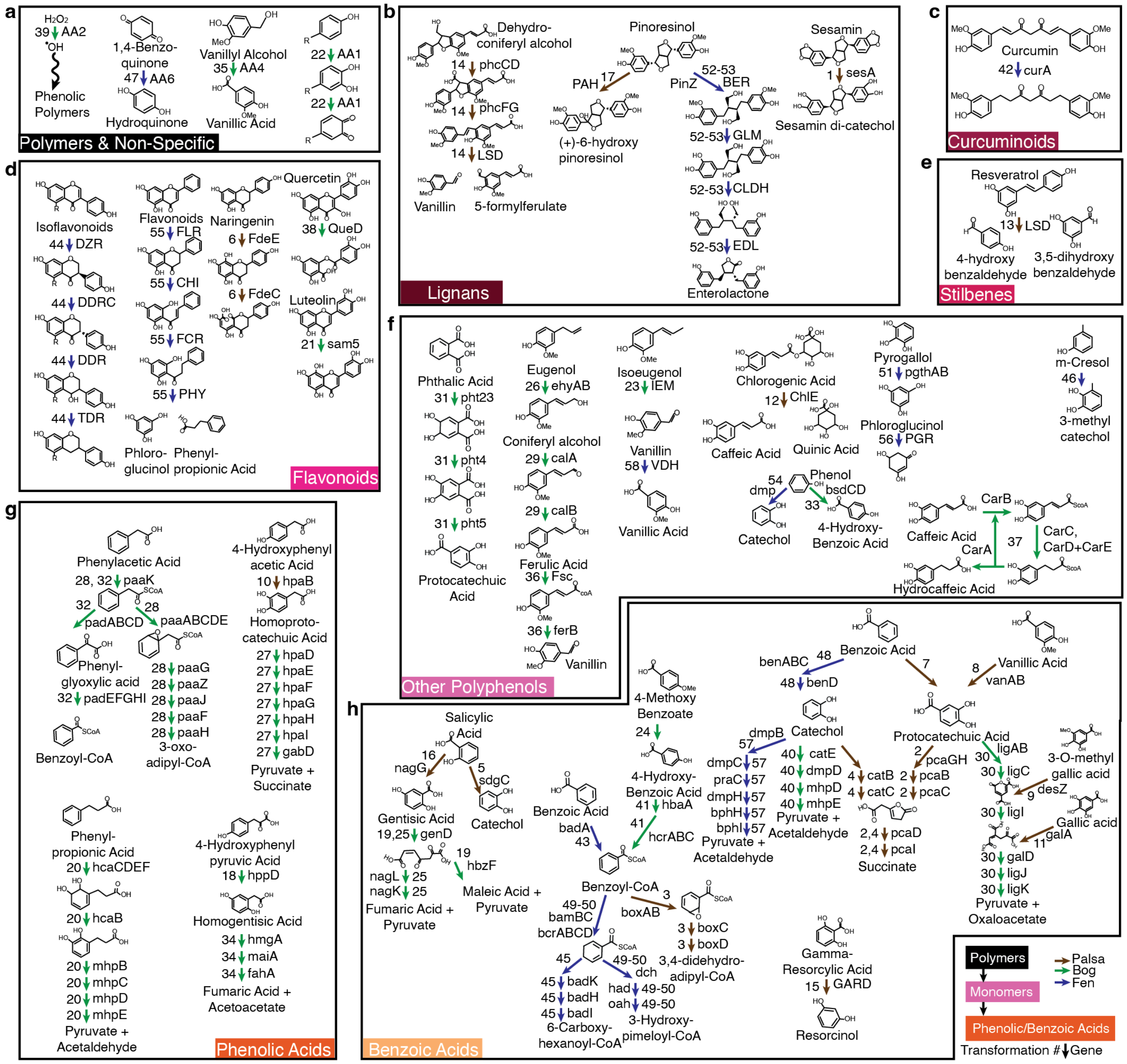

Supplementary Figure 6. The polyphenol transformations observed in the Stordalen Mire metatranscriptome. Transformations are shown by polyphenol type: (**a**) polymers and non-specific reactions, (**b**) lignans, (**c**) curcuminoids, (**d**) flavonoids, (**e**) stilbenes, (**f**) other polyphenols, (**g**) phenolic acids and (**h**) benzoic acids. In the reaction schemes, numbers correspond to transformations from **Fig. 2**; see **Supplementary Data 2** for more detail on each transformation. Arrow colors correspond to the habitat cluster for the transformation: palsa (brown), bog (green), and fen (blue). In #39, the wavy arrow indicates the hydroxyl radical diffuses away to act on phenolic polymers.

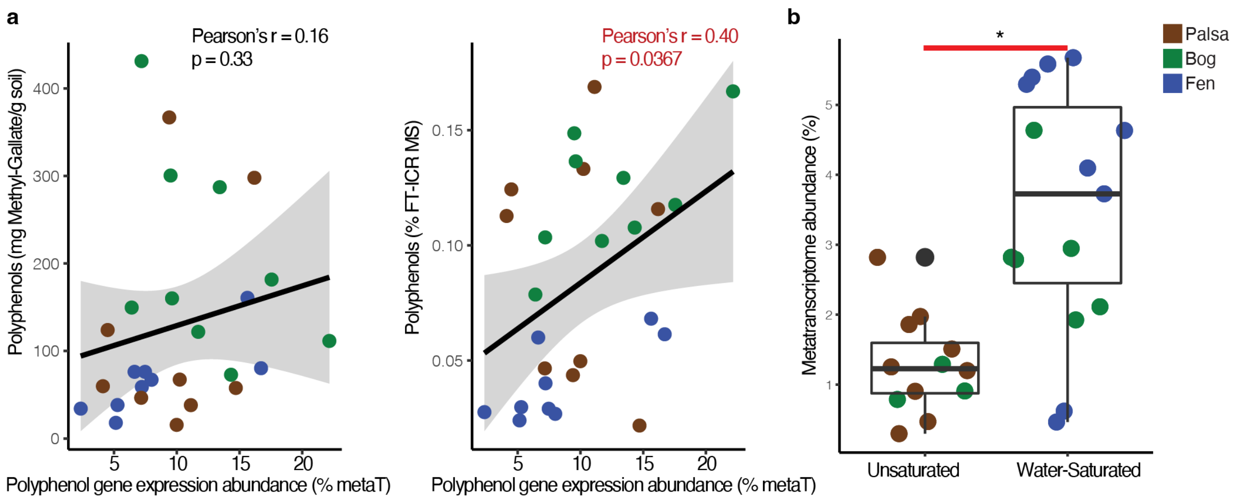

Supplementary Figure 7. Relative expression of anoxic polyphenol transformations in unsaturated and water saturated samples. (**a**) Relationship between the relative metatranscriptome abundance of the 58 polyphenol transformation pathways and polyphenol content by the Folin-Ciocalteu Assay (left, n=27) and FT-ICRMS (right, n=27). The black lines represent the linear trendlines across the data points, and the grey shaded regions correspond to the 95% confidence interval. (**b**) Relative metatranscriptome abundance of the anoxic polyphenol transformation pathways in unsaturated (left, n=12 biologically independent samples) is significantly less than in saturated (right, n=15 biologically independent samples) samples (one-way ANOVA, p=0.000294). The lower and upper boxplot edges represent the 25th and 75th percentiles, respectively, and the middle line is the median. The whiskers extend from the median by 1.5× the interquartile range. Black data points represent outliers. In both, datapoints are colored by the habitat.

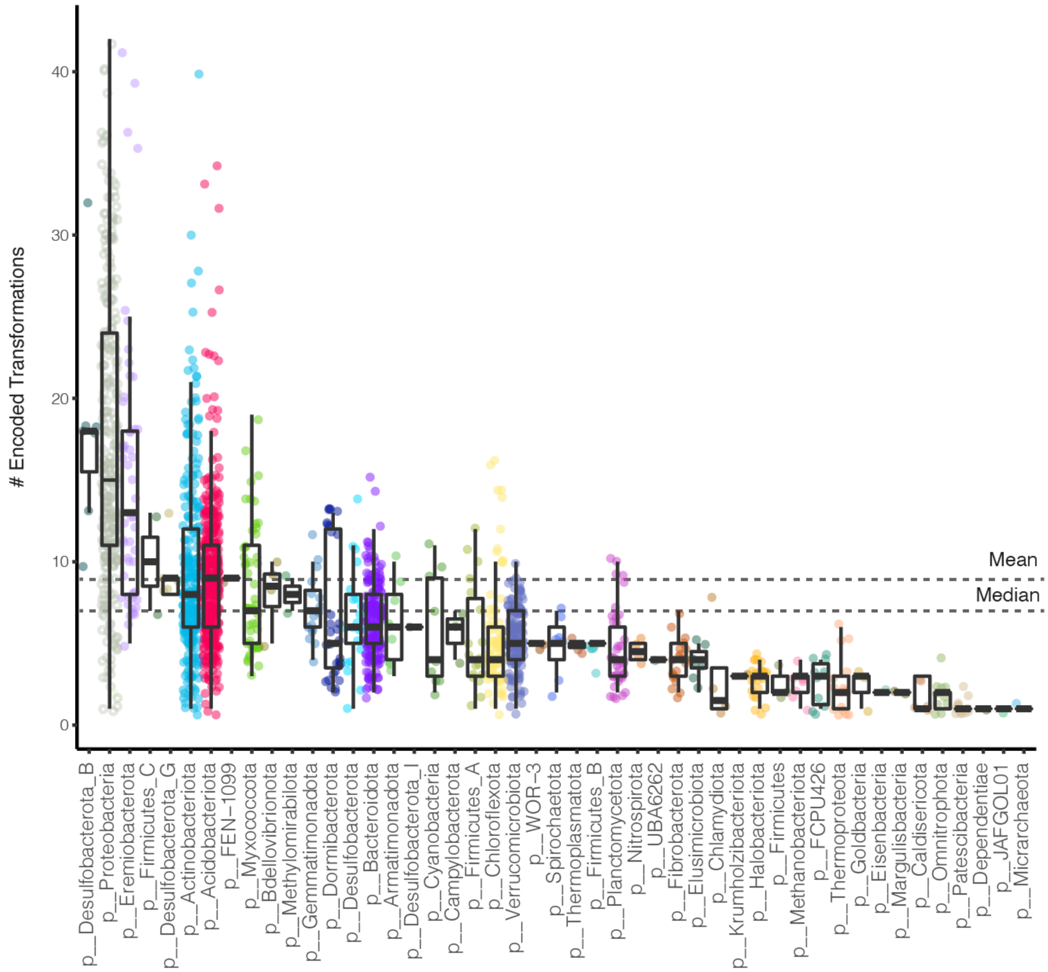

Supplementary Figure 8. MAG Polyphenol transformations encoded by phylum. Each point represents a MAG in a given phylum, with point color corresponding to phylum. Overlaid boxplots show the distribution of encoded transformations across the phylum. The lower and upper boxplot edges represent the 25th and 75th percentiles, respectively, and the middle line is the median. The whiskers extend from the median by 1.5× the interquartile range. The mean and median values across all MAGs are marked at 9 and 7, respectively.

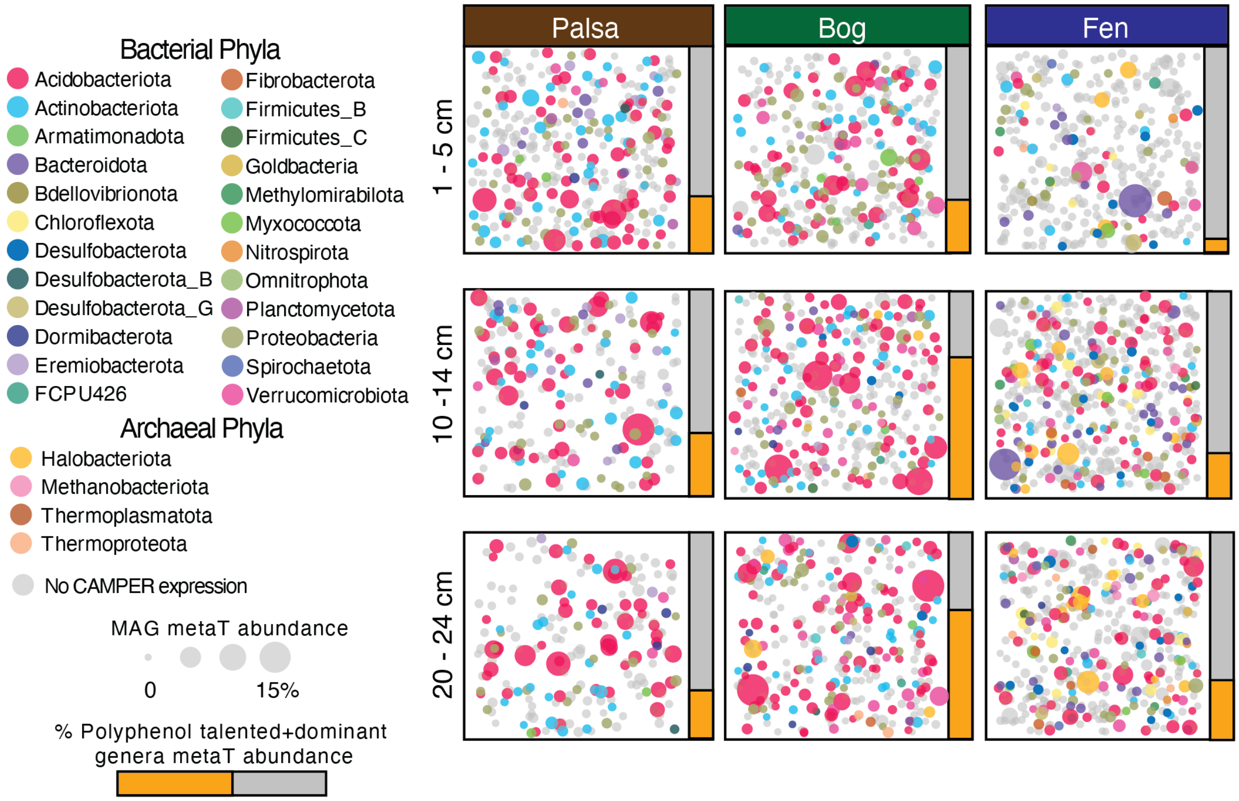

Supplementary Figure 9. Stordalen Mire microbiomes express polyphenol transformation genes in field metatranscriptomes. The average abundance of metagenome-assembled genomes (MAGs, n=3) in field metatranscriptomes in the palsa (left column), bog (middle column), and fen (right column), in surface (1-5cm, top row), middle (10-14cm, middle row), and deep (20-24cm, bottom row) depths. Each circle represents a MAG and is sized by the mean relative abundance in each habitat and depth. Grey circles represent MAGs not expressing CAMPER genes, while circles are colored according to phylum for MAGs expressing at least one CAMPER transformation in each site and depth. MAG abundance data can be found in numeric form in **Supplementary Data 3**, and MAG polyphenol gene expression by site and depth can be found in **Supplementary Data 2**. The bar plot at right of each habitat and depth shows (in orange) the average relative abundance of MAGs belonging the five talented and dominant genera noted in **Fig. 3**.

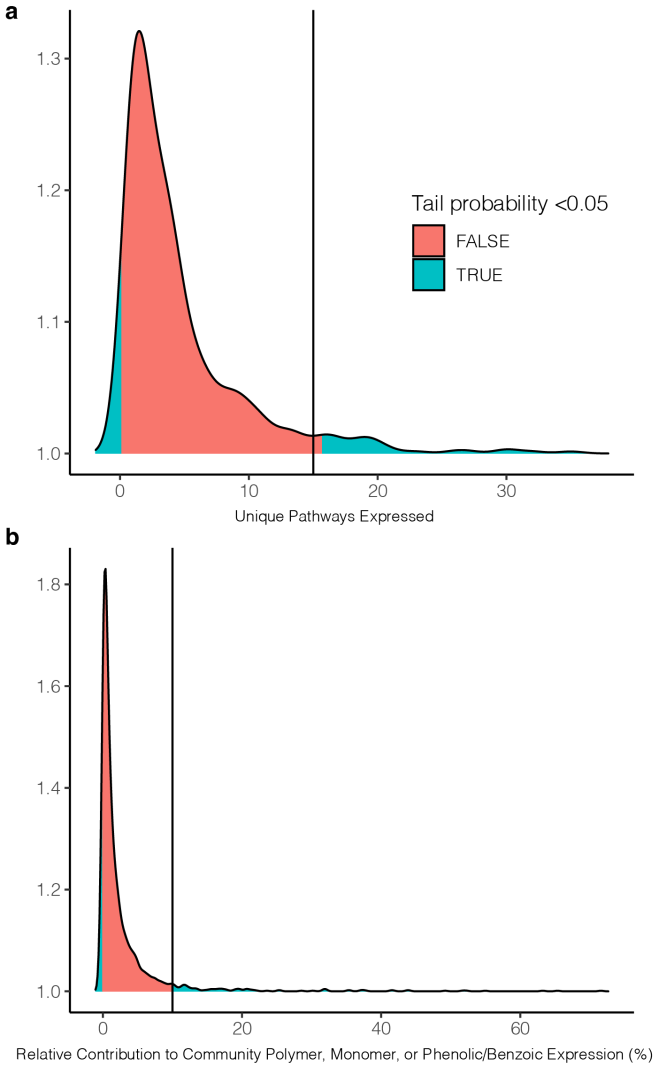

Supplementary Figure 10. Thresholds for polyphenol talent and dominance. (A) The empirical density function curve of the number of unique polyphenol-active pathways expressed per genus in each habitat. The vertical line at 15 represents the threshold chosen for polyphenol talent, corresponding to the 95% probability. (**B**) The empirical density function curve of the relative contribution of each genus to polymeric, monomeric, or phenolic/benzoic active gene expression in each habitat and depth. The vertical line at 10% represents the threshold chosen for polyphenol dominance, corresponding to the 95% probability.

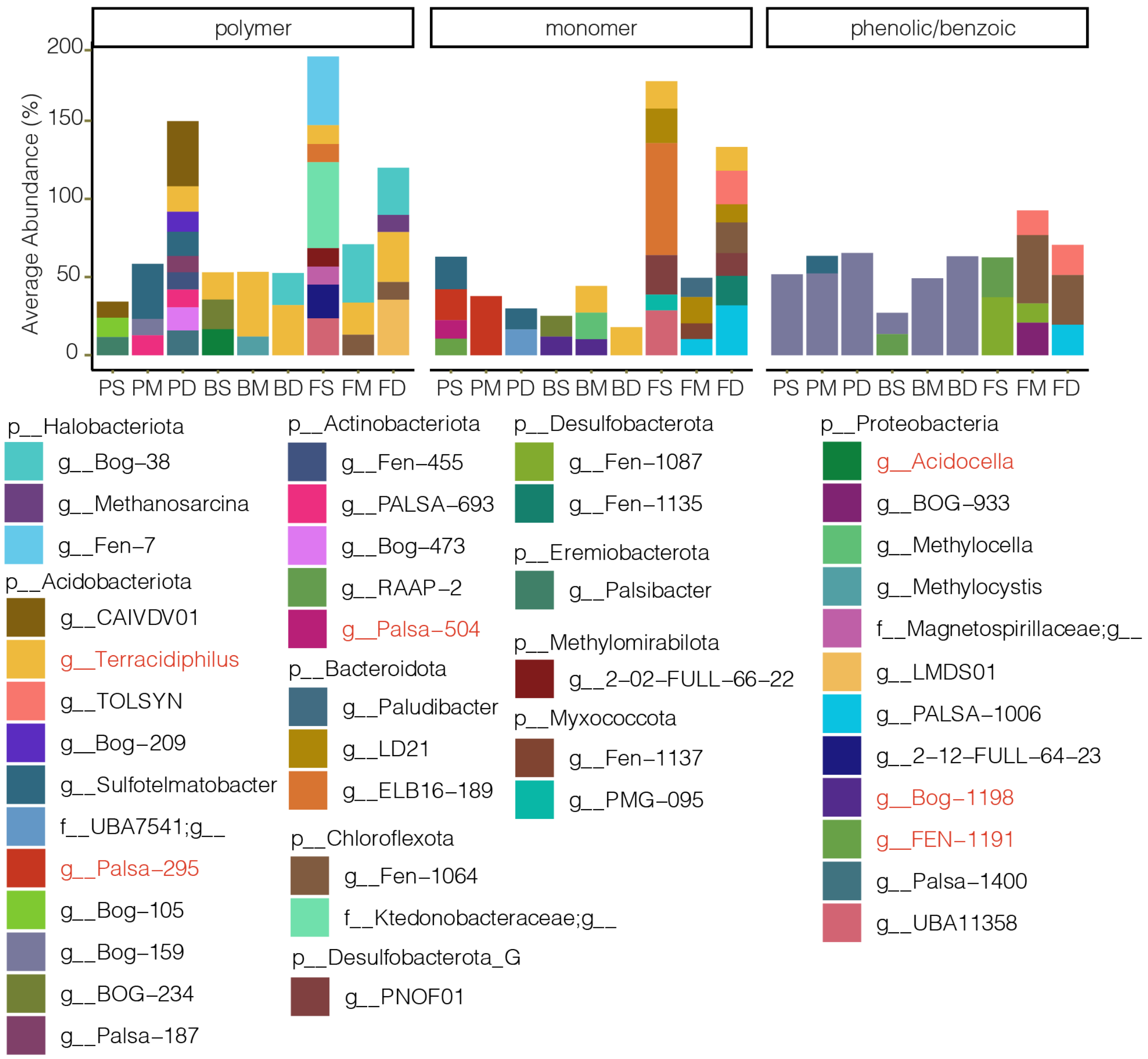

Supplementary Figure 11. Relative contribution of microbial genera to polyphenol transformation transcription. The average contribution of polyphenol dominant genera to polymeric polyphenol (left), monomeric polyphenol (middle), and phenolic/benzoic acid (right) transformation gene expression. Genera contributing on average ≥10% of expression within a site and depth are shown (n=3). Names in red denote the dominant and talented genera. Sample groups are shown on the x-axis for palsa (P), bog (B), and fen (F) sites at surface (S), middle (M), and deep (D) depths (for example, BM is bog middle).

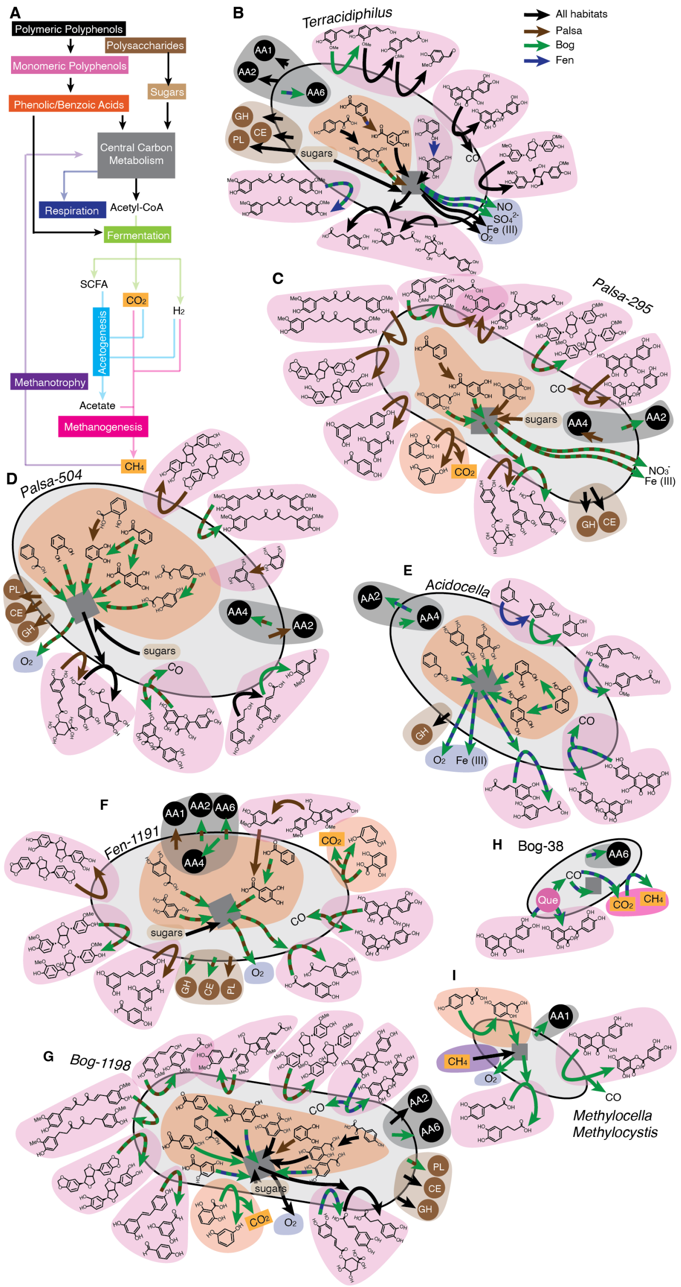

Supplementary Figure 12. **Polyphenol transformations carried out by important Stordalen Mire genera.** (**A**) Polyphenol transformations can feed to central carbon metabolism or serve as terminal electron acceptors for fermentation. Polyphenol transformations and broader metabolism by MAGs in the (**B**) *Terracidiphilus*, (**C**) Palsa-295, (**D**) *Palsa-504*, (**E**) *Acidocella*, (**F**) Fen-1191, (**G**) *Bog-1198*, (**H**) Bog-38, (**I**) *Methylocella* and *Methylocystis*. Displayed reactions are shaded by their place in the carbon cycle in (A). Arrows indicate habitat where function was expressed. Abbreviations are as follows: glycoside hydrolase (GH), carbohydrate esterase (CE), polysaccharide lyase (PL), PO (AA1), peroxidase (AA2), vanillyl-alcohol oxidase (AA4). Grey boxed in each cartoon represents central carbon metabolism. For data behind this figure, see **Supplementary Data 5**.

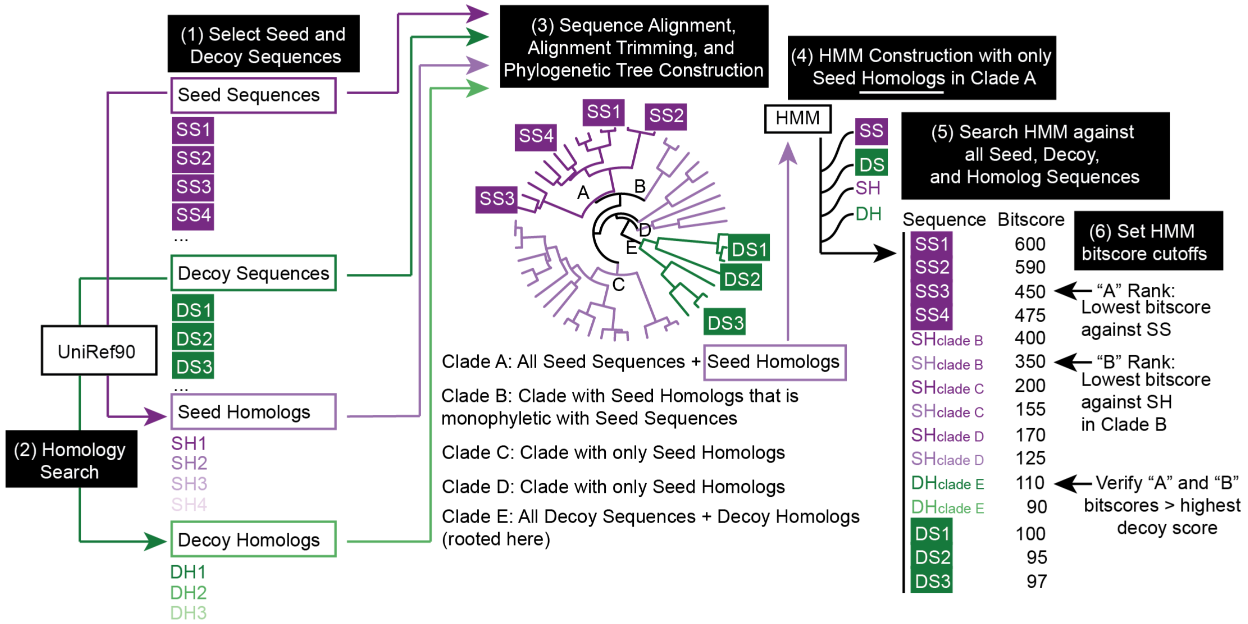

Supplementary Figure 13. Workflow for CAMPER Hidden-Markov Model (HMM) construction and score threshold designation. For each functional gene of interest, we BLAST'ed sequences of biochemically characterized genes ("seed sequences", SS) against UniRef90 and pulled the top 200 hits for each seed (resulting in "seed homologs", SH). In parallel, we BLAST'ed sequences of homologous but functionally distinct genes ("decoy sequences", DS) against UniRef90 and pulled the top 200 hits for each decoy (resulting in "decoy homologs", DH). We then created and trimmed sequence alignments of the seed sequences, seed homologs, decoy sequences, and decoy homologs, and used this alignment to construct a phylogenetic tree. We rooted the tree on clades that contained all decoy sequences and homologs (clade E). We then picked out the clade the contained all seed sequences (clade A) and pulled only the seed homolog sequences from this clade to build the HMM. Importantly, we left the seed sequences out of HMM construction as a hold-out dataset. Once the HMM was constructed, we searched the HMM against all seed sequences, seed homologs, decoy sequences, and decoy homologs. We designated two scores for each HMM: the "A" score was the lowest bitscore of the alignment of the HMM against a seed sequence (in the figure above, A score = 450). As the seed sequences were not used in HMM construction, this cut-off represents a trusted score, aimed to increase the true positive rate. When possible, a "B" score was designated using the lowest bitscore obtained from aligning the HMM against a seed homolog in a clade monophyletic to the seed sequences (clade B, in the figure above, A score = 350). Importantly, we verified the A and B scores were greater than the highest bitscore obtained from alignments of the HMM with a decoy sequence (in the figure above, highest decoy score = 110).

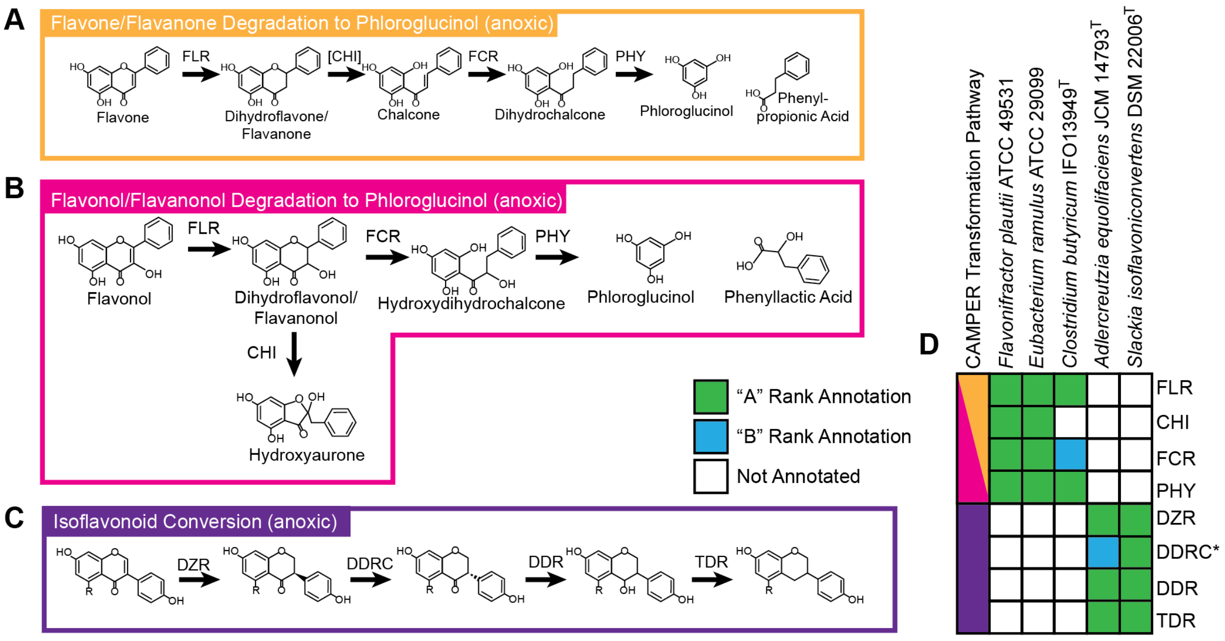

E

	A Rank Only	A+B Rank
Precision	1	1
Recall	0.89	1

$$\text{Precision} = \frac{\text{True Positives}}{\text{True+False Positives}} \qquad \text{Recall} = \frac{\text{True Positives}}{\text{True Positives+False Negatives}}$$

Supplementary Figure 14. **Validation of CAMPER annotations against five experimentally characterized isolate genomes.** Genomes from 5 isolates with experimental evidence for (A) flavone/flavanone degradation (yellow), (B) flavonol/flavanonol degradation (pink), or (C) isoflavonoid conversion (purple) were annotated with CAMPER. In CAMPER, the genes spanning these pathways are covered by 7 HMMs and 1 BLAST search (DDRC, marked with *). The genes for flavone/flavanone degradation and flavonol/flavanonol degradation overlap, with the note that CHI is optional in some flavanone degradation pathways (noted with [CHI]). (D) The presence of A rank annotations in the genomes is noted with green, and B rank annotations are noted in blue. Collectively, CAMPER provides annotations that match the experimental phenotypes for these 5 isolates. (E) Using the isolate genome annotations, precision and recall were calculated using A rank annotations alone, and both A and B rank annotations. See **Methods** for genome accession numbers, and **Supplementary Data 2** for CAMPER annotation outputs.

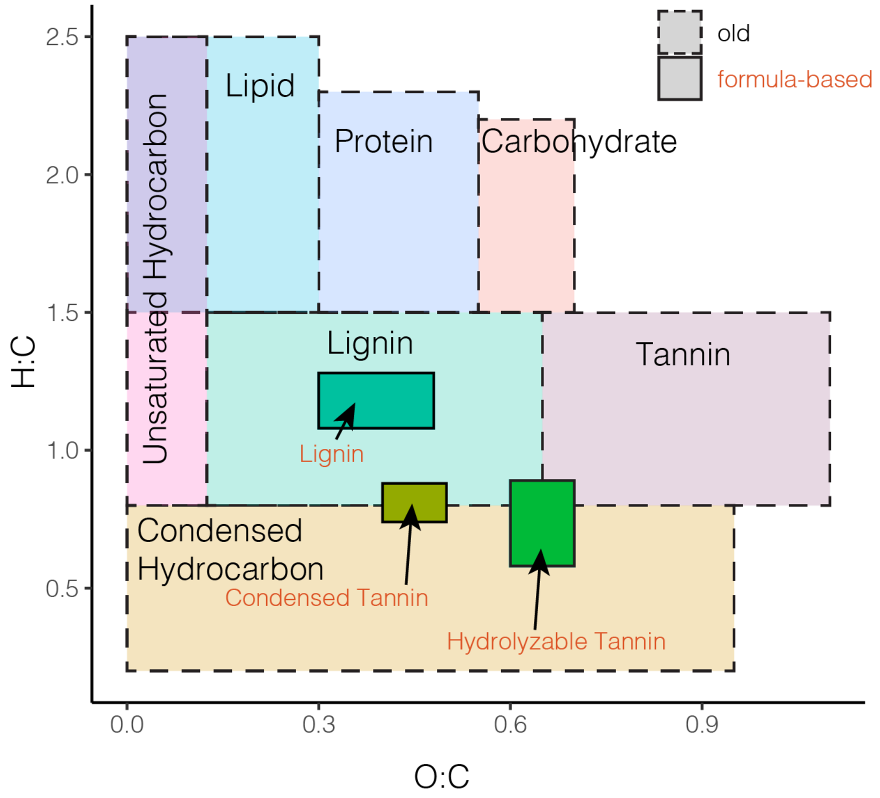

Supplementary Figure 15. **Van Krevlen boundaries for lignin, condensed tannins, and hydrolyzable tannins proposed in this study.** Classic boundaries are shown with dotted lines. New boundaries established using known formulae for lignin and condensed and hydrolyzable tannins are shown with solid black lines, and red labels. See **Supplementary Data 6** for data underlying these boundaries.

SOM Tables

Supplementary Table 1. Genes encoding polyphenol-active enzymes identified from cross-microbiome search used for custom annotations.

Gene	Name	Substrate	Oxygen	Annotation Type	Phyla represented (# genera) in seed sequences	Refs.
FLR	Flavone/flavonol reductase	Flavonoids	Anoxic	HMM	Actinomycetota (1), Bacillota_A (2), Fusobacteriota (1), Spirochaetota (1), Ascomycota (3)	[5]
CHI	Chalcone isomerase	Flavonoids	Anoxic	HMM	Acidobacteriota (1), Bacillota (1), Bacillota_A (11)	[6,7]
FCR	Flavanone/ flavanonol-cleaving reductase	Flavonoids	Anoxic	HMM	Acidobacteriota (1), Bacillota_A (4), Bacillota_C (3)	[8]
PHY	Phloretin Hydrolase	Flavonoids	Anoxic	HMM	Acidobacteriota (1), Actinomycetota (1), Bacillota_A (2), Bacillota_C (2), Halobacteriota (1)	[9–11]
DZR	daidzein reductase	Isoflavonoids	Anoxic	HMM	Actinomycetota (2), Bacillota (1)	[12–15]
DDR	dihydrodaidzein reductase	Isoflavonoids	Anoxic	HMM	Actinomycetota (2), Bacillota (1)	[13–16]
DDRC	dihydrodaidzein racemase	Isoflavonoids	Anoxic	BLAST	Bacillota (1)	[17]
TDR	tetrahydrodaidzein reductase	Isoflavonoids	Anoxic	HMM	Actinomycetota (3), Bacillota (1)	[13–16]
LSD	Iignostilbene dioxygenase	Stilbenes, lignans	Oxic	HMM	Pseudomonadota (3)	[18,19]
SesA	THF-dependent sesamin/sesamin-monocatechol methylenetransfera se	Lignan	Anoxic	BLAST	Actinomycetota (1)	[20]
BER	benzyl ether reductase	Lignan	Anoxic	BLAST	Actinomycetota (1)	[21]
GLM	guaiacol lignan methyltransferase	Lignan	Anoxic	BLAST	Bacillota_A (1)	[21]
CLDH	catechol lignan dehydroxylase	Lignan	Anoxic	BLAST	Actinomycetota (1)	[21]
EDL	END lactonizing enzyme	Lignan	Anoxic	BLAST	Bacillota_A (1)	[21]
PinZ	pinoresinol reductase	Lignan	Anoxic	BLAST	Pseudomonadota (2)	[22]
CurA	curcumin/ dihydrocurcumin reductase	Curcuminoids	Anoxic	BLAST	Pseudomonadota (1)	[23]
CarA	hydrocaffeyl-CoA:caffeate CoA transferase	Caffeic Acid	Anoxic	BLAST	Bacillota_A (1)	[24]
CarB	caffeyl-CoA synthetase	Caffeic Acid	Anoxic	BLAST	Bacillota_A (1)	[24]

CarC	caffeyl-CoA reductase	Caffeic Acid	Anoxic	BLAST	Bacillota_A (1)	[25]
CarD	electron transfer flavoprotein A	Caffeic Acid	Anoxic	BLAST	Bacillota_A (1)	[26]
CarE	electron transfer flavoprotein B	Caffeic Acid	Anoxic	BLAST	Bacillota_A (1)	[26]
PgthAB	pyrogallol-phloroglucinol transhydroxylase	Pyrogallol	Anoxic	BLAST	Desulfobacterota_F (1)	[27]
PGR	phloroglucinol reductase	Phloroglucinol	Anoxic	BLAST	Bacillota_A (1)	[28]
EhyAB	eugenol hydroxylase	Eugenol	Oxic	BLAST	Pseudomonadota (1)	[29,30]
CalA	coniferyl alcohol dehydrogenase	Coniferyl Alcohol	Oxic	BLAST	Pseudomonadota (1)	[31]
CalB	coniferyl aldehyde dehydrogenase	Coniferyl Aldeyhde	Oxic	BLAST	Pseudomonadota (1)	[32]
FCS	feruloyl-CoA synthetase	Ferulic Acid	Oxic	BLAST	Pseudomonadota (1)	[33]
IEM	isoeugenol monooxygenase	Isoeugenol	Oxic	BLAST	Pseudomonadota (1)	[34]
PhcC	(+)-dehydrodiconiferyl acid dehydrogenase	Lignan	Oxic	BLAST	Pseudomonadota (1)	[35]
PhcD	(-)-dehydrodiconiferyl acid dehydrogenase/ hydroxylase	Lignan	Oxic	BLAST	Pseudomonadota (1)	[35]
PhcF	(+)-DCA-CC decarboxylase	Lignan	Oxic	BLAST	Pseudomonadota (1)	[36]
PhcG	(-)-DCA-CC decarboxylase	Lignan	Oxic	BLAST	Pseudomonadota (1)	[36]
Pah	pinoresinol-a-hydroxylase	Lignan	Anoxic	BLAST	Pseudomonadota (1)	[37]
ChlE	chlorogenic acid esterase	Chlorogenic Acid	Oxic	BLAST	Bacillota (1) Pseudomonadota (1)	[38–40]
FdeE	naringenin hydroxylase	Flavonoid	Oxic	BLAST	Pseudomonadota (1)	[41]
FdeC	naringenin dioxygenase	Flavonoid	Oxic	BLAST	Pseudomonadota (1)	[41]
QueD	quercetin-2,3-dioxygenase	Flavonoid	Oxic	BLAST	Actinomycetota (1), Bacillota (1)	[42,43]
Sam5	flavonoid hydroxylase	Flavonoid	Oxic	BLAST	Actinomycetota (1)	[44]
GARD	Gamma-resorcylic acid decarboxylase	Resorcylic Acid	Oxic	BLAST	Pseudomonadota (1)	[45]

Supplementary References

1. Sinsabaugh, R. L. Phenol oxidase, peroxidase and organic matter dynamics of soil. *Soil Biology and Biochemistry* **42**, 391–404 (2010).
2. Freeman, C., Ostle, N. & Kang, H. An enzymic 'latch' on a global carbon store: A shortage of oxygen locks up carbon in peatlands by restraining a single enzymes. *Nature* **409**, 149 (2001).
3. Fenner, N. & Freeman, C. Drought-induced carbon loss in peatlands. *Nature Geoscience* **4**, 895–900 (2011).
4. Fenner, N. & Freeman, C. Woody litter protects peat carbon stocks during drought. *Nature Climate Change* **10**, 363–369 (2020).
5. Yang, G. *et al.* Discovery of an ene-reductase for initiating flavone and flavonol catabolism in gut bacteria. *Nat Commun* **12**, 790 (2021).
6. Braune, A. *et al.* Chalcone isomerase from Eubacterium ramulus catalyzes the ring contraction of flavanonols. *Journal of Bacteriology* **198**, 2965–2974 (2016).
7. Meinert, H. *et al.* Discovery of Novel Bacterial Chalcone Isomerases by a Sequence-Structure-Function-Evolution Strategy for Enzymatic Synthesis of (S)-Flavanones. *Angewandte Chemie International Edition* **60**, 16874–16879 (2021).
8. Braune, A., Gütschow, M. & Blauta, M. An NADH-dependent reductase from Eubacterium ramulus catalyzes the stereospecific heteroring cleavage of flavanones and flavanonols. *Applied and Environmental Microbiology* **85**, 1233–1252 (2019).
9. Schoefer, L., Braune, A. & Blaut, M. Cloning and expression of a phloretin hydrolase gene from Eubacterium ramulus and characterization of the recombinant enzyme. *Applied and Environmental Microbiology* **70**, 6131–6137 (2004).

10. Han, J. *et al.* Discovery and structural analysis of a phloretin hydrolase from the opportunistic human pathogen *Mycobacterium abscessus*. *The FEBS Journal* **286**, 1959–1971 (2019).

11. Rodriguez-Castaño, G. P., Rey, F. E., Caro-Quintero, A. & Acosta-González, A. Gut-derived Flavonifractor species variants are differentially enriched during in vitro incubation with quercetin. *PLoS One* **15**, e0227724 (2020).

12. Shimada, Y. *et al.* Cloning and Expression of a Novel NADP(H)-Dependent Daidzein Reductase, an Enzyme Involved in the Metabolism of Daidzein, from Equol-Producing Lactococcus Strain 20-92. *Applied and Environmental Microbiology* **76**, 5892–5901 (2010).

13. Tsuji, H., Moriyama, K., Nomoto, K. & Akaza, H. Identification of an Enzyme System for Daidzein-to-Equol Conversion in Slackia sp. Strain NATTS. *Applied and Environmental Microbiology* **78**, 1228–1236 (2012).

14. Schröder, C., Matthies, A., Engst, W., Blaut, M. & Braune, A. Identification and Expression of Genes Involved in the Conversion of Daidzein and Genistein by the Equol-Forming Bacterium Slackia isoflavoniconvertens. *Applied and Environmental Microbiology* **79**, 3494–3502 (2013).

15. Kawada, Y., Yokoyama, S., Yanase, E., Niwa, T. & Suzuki, T. The production of *S*-equol from daidzein is associated with a cluster of three genes in *Eggerthella* sp. YY7918. *Bioscience of Microbiota, Food and Health* **35**, 113–121 (2016).

16. Shimada, Y. *et al.* Identification of Two Novel Reductases Involved in Equol Biosynthesis in Lactococcus Strain 20–92. *MIP* **21**, 160–172 (2011).

17. Shimada, Y. *et al.* Identification of a Novel Dihydrodaidzein Racemase Essential for Biosynthesis of Equol from Daidzein in Lactococcus sp. Strain 20-92. *Appl Environ Microbiol* **78**, 4902–4907 (2012).

18. Kamoda, S. & Saburi, Y. Cloning, Expression, and Sequence Analysis of a Lignostilbene-α,β-dioxygenase Gene from Pseudomonas paucimobilis TMY1009. *Bioscience, Biotechnology, and Biochemistry* **57**, 926–930 (1993).

19. Loewen, P. C. *et al.* Structure and function of a lignostilbene-α,β-dioxygenase orthologue from Pseudomonas brassicacearum. *BMC Biochemistry* **19**, 8 (2018).

20. Kumano, T., Fujiki, E., Hashimoto, Y. & Kobayashi, M. Discovery of a sesamin-metabolizing microorganism and a new enzyme. *Proceedings of the National Academy of Sciences* **113**, 9087–9092 (2016).

21. Bess, E. N. *et al.* Genetic basis for the cooperative bioactivation of plant lignans by Eggerthella lenta and other human gut bacteria. *Nat Microbiol* **5**, 56–66 (2020).

22. Fukuhara, Y. *et al.* Discovery of pinoresinol reductase genes in sphingomonads. *Enzyme and Microbial Technology* **52**, 38–43 (2013).

23. Hassaninasab, A., Hashimoto, Y., Tomita-Yokotani, K. & Kobayashi, M. Discovery of the curcumin metabolic pathway involving a unique enzyme in an intestinal microorganism. *Proceedings of the National Academy of Sciences* **108**, 6615–6620 (2011).

24. Hess, V., Vitt, S. & Müller, V. A Caffeyl-Coenzyme A Synthetase Initiates Caffeate Activation prior to Caffeate Reduction in the Acetogenic Bacterium Acetobacterium woodii. *J Bacteriol* **193**, 971–978 (2011).

25. Bertsch, J., Parthasarathy, A., Buckel, W. & Müller, V. An Electron-bifurcating Caffeyl-CoA Reductase. *J Biol Chem* **288**, 11304–11311 (2013).

26. Imkamp, F., Biegel, E., Jayamani, E., Buckel, W. & Müller, V. Dissection of the Caffeate Respiratory Chain in the Acetogen Acetobacterium woodii: Identification of an Rnf-Type NADH Dehydrogenase as a Potential Coupling Site. *Journal of Bacteriology* **189**, 8145–8153 (2007).

27. Messerschmidt, A. *et al.* Crystal structure of pyrogallol–phloroglucinol transhydroxylase, an Mo enzyme capable of intermolecular hydroxyl transfer between phenols. *Proceedings of the National Academy of Sciences* **101**, 11571–11576 (2004).

28. Haddock, J. D. & Ferry, J. G. Purification and properties of phloroglucinol reductase from Eubacterium oxidoreducens G-41. *The Journal of biological chemistry* **264**, 4423–4427 (1989).

29. Brandt, K., Thewes, S., Overhage, J., Priefert, H. & Steinbüchel, A. Characterization of the eugenol hydroxylase genes (ehyA/ehyB) from the new eugenol-degrading Pseudomonas sp. strain OPS1. *Appl Microbiol Biotechnol* **56**, 724–730 (2001).

30. Priefert, H., Overhage, J. & Steinbüchel, A. Identification and molecular characterization of the eugenol hydroxylase genes (ehyA/ehyB) of Pseudomonas sp. strain HR199. *Arch Microbiol* **172**, 354–363 (1999).

31. Overhage, J., Steinbüchel, A. & Priefert, H. Biotransformation of eugenol to ferulic acid by a recombinant strain of Ralstonia eutropha H16. *Appl Environ Microbiol* **68**, 4315–4321 (2002).

32. Achterholt, S., Priefert, H. & Steinbüchel, A. Purification and Characterization of the Coniferyl Aldehyde Dehydrogenase from *Pseudomonas* sp. Strain HR199 and Molecular Characterization of the Gene. *Journal of Bacteriology* **180**, 4387–4391 (1998).

33. Masai, E. *et al.* Cloning and Characterization of the Ferulic Acid Catabolic Genes of Sphingomonas paucimobilis SYK-6. *Applied and Environmental Microbiology* **68**, 4416–4424 (2002).

34. Ryu, J.-Y. *et al.* Isoeugenol monooxygenase and its putative regulatory gene are located in the eugenol metabolic gene cluster in Pseudomonas nitroreducens Jin1. *Arch Microbiol* **192**, 201–209 (2010).

35. Takahashi, K. *et al.* Membrane-Associated Glucose-Methanol-Choline Oxidoreductase Family Enzymes PhcC and PhcD Are Essential for Enantioselective Catabolism of Dehydrodiconiferyl Alcohol. *Applied and Environmental Microbiology* **81**, 8022–8036 (2015).

36. Takahashi, K., Miyake, K., Hishiyama, S., Kamimura, N. & Masai, E. Two novel decarboxylase genes play a key role in the stereospecific catabolism of dehydrodiconiferyl alcohol in Sphingobium sp. strain SYK-6. *Environmental Microbiology* **20**, 1739–1750 (2018).

37. Shettigar, M. *et al.* Oxidative Catabolism of (+)-Pinoresinol Is Initiated by an Unusual Flavocytochrome Encoded by Translationally Coupled Genes within a Cluster of (+)-Pinoresinol-Coinduced Genes in Pseudomonas sp. Strain SG-MS2. *Appl Environ Microbiol* **86**, e00375-20 (2020).

38. Yao, J., Gui, L. & Long, Q. A chlorogenic acid esterase from a metagenomic library with unique substrate specificity and its application in caffeic and ferulic acid production from agricultural byproducts. *Biocatalysis and Biotransformation* **40**, 413–421 (2022).

39. Smith, M. A., Weaver, V. B., Young, D. M. & Ornston, L. N. Genes for chlorogenate and hydroxycinnamate catabolism (hca) are linked to functionally related genes in the dca-pca-

qui-pob-hca chromosomal cluster of Acinetobacter sp. strain ADP1. *Appl Environ Microbiol* **69**, 524–532 (2003).

40. Wang, X., Bai, Y., Cai, Y. & Zheng, X. Biochemical characteristics of three feruloyl esterases with a broad substrate spectrum from Bacillus amyloliquefaciens H47. *Process Biochemistry* **53**, 109–115 (2017).

41. Marin, A. M. *et al.* Naringenin degradation by the endophytic diazotroph Herbaspirillum seropedicae SmR1. *Microbiology* **159**, 167–175 (2013).

42. Bowater, L., Fairhurst, S. A., Just, V. J. & Bornemann, S. *Bacillus subtilis* YxaG is a novel Fe-containing quercetin 2,3-dioxygenase. *FEBS Letters* **557**, 45–48 (2004).

43. Merkens, H., Kappl, R., Jakob, R. P., Schmid, F. X. & Fetzner, S. Quercetinase QueD of Streptomyces sp. FLA, a Monocupin Dioxygenase with a Preference for Nickel and Cobalt. *Biochemistry* **47**, 12185–12196 (2008).

44. Lee, H., Kim, B.-G. & Ahn, J.-H. Production of bioactive hydroxyflavones by using monooxygenase from Saccharothrix espanaensis. *Journal of Biotechnology* **176**, 11–17 (2014).

45. Iwasaki, Y., Kino, K., Nishide, H. & Kirimura, K. Regioselective and enzymatic production of γ-resorcylic acid from resorcinol using recombinant Escherichia coli cells expressing a novel decarboxylase gene. *Biotechnol Lett* **29**, 819–822 (2007).
